# Mn Doping Enhances the Antibacterial, Antibiofilm and Anti-Virulence Activity of ZnO Nanoparticles

**DOI:** 10.3390/nano16161019

**Published:** 2026-08-18

**Authors:** Dario Morganti, Domenico Franco, Giuseppe Nicotra, Elena Spagnoli, Stefano Zampolli, Vittorio Morandi, Sabrina Conoci

**Affiliations:** 1 National Research Council, Institute for Nanostructured Materials (CNR-ISMN), Bologna Section, Via Piero Gobetti 101, 40129 Bologna, Italy; elena.spagnoli@cnr.it (E.S.); stefano.zampolli@cnr.it (S.Z.); vittorio.morandi@cnr.it (V.M.); sabrina.conoci@unibo.it (S.C.); 2 Department of Chemical, Biological, Pharmaceutical and Environmental Sciences, University of Messina, Viale Ferdinando Stagno d’Alcontres 31, 98166 Messina, Italy; 3 National Research Council, Institute for Microelectronics and Microsystems (CNR-IMM), 95121 Catania, Italy; giuseppe.nicotra@cnr.it; 4 Department of Chemistry G. Ciamician, University of Bologna, Via Piero Gobetti 85, 40129 Bologna, Italy

**Keywords:** nanomaterials, ZnO nanoparticles, Mn doping, chemical characterization, antibacterial activity, biofilm

## Abstract

The modulation of ZnO physicochemical properties through Mn doping represents a promising strategy for the development of multifunctional nanomaterials with enhanced biological performance. In this study, we investigate how the nominal Mn concentration influences both the physicochemical properties and antibacterial, antibiofilm, and anti-virulence activities of ZnO nanoparticles (NPs). Mn-doped ZnO nanoparticles containing nominal Mn from 2.5 to 10 mol% were synthesized through a simple wet-chemical approach and characterized by UV–Vis, Raman, TEM, EDX, and EELS analyses. The resulting ZnO-based NPs showed average dimensions of 3.7–4.8 nm, while increasing Mn incorporation produced measurable changes in optical response and morphology of nanoparticles. Antibacterial activity was evaluated against Gram-positive and Gram-negative bacterial models by assessing planktonic growth inhibition, biofilm formation, and pyocyanin production. The sample with the highest Mn amount (Mn_10_-ZnO) markedly enhanced antibacterial performance by reducing MIC_90_ from 150 to 37.5 μg/mL against *Staphylococcus aureus* and from 300 to 75 μg/mL for *Pseudomonas aeruginosa*. Mn doping also enhanced biofilm inhibition and produced a progressive reduction in pyocyanin synthesis. These results establish a concentration-dependent relationship between Mn concentration, nanoparticle properties, and antibacterial performance, highlighting the potential of Mn-doped ZnO nanoparticles for the development of anti-infective biomaterials, including antimicrobial coatings for implantable medical devices.

## 1. Introduction

Bacterial infections associated with biomaterials and implantable medical devices still represent one of the most important challenges in modern healthcare. The colonization of surfaces by pathogenic microorganisms and the subsequent formation of bacterial biofilms can severely compromise the long-term performance of biomedical devices, often leading to chronic infections, implant failure, tissue damage, and prolonged antibiotic therapies. This issue is particularly relevant in orthopedic applications, where bacterial adhesion onto biomaterial surfaces may trigger persistent infections that are extremely difficult to eradicate due to the intrinsic resistance of biofilms to both antibiotics and host immune responses [1,2,3].

The rapid diffusion of antibiotic-resistant bacterial strains has stimulated the development of alternative antimicrobial approaches able to prevent bacterial colonization without promoting resistance phenomena [4,5]. In this scenario, nanostructured materials have attracted increasing interest as promising platforms for the development of next-generation antibacterial systems owing to their unique physicochemical properties and multifunctional behavior at the nanoscale [6,7,8]. In particular, the possibility of tailoring the physicochemical properties of nanostructures through controlled synthesis, surface engineering, and doping strategies has opened new perspectives for the development of multifunctional nanosystems for both technological and biomedical applications [9,10,11]. In this context, inorganic metal oxide nanostructures have emerged as particularly promising systems because of their intrinsic antibacterial activity, chemical stability, and long-term durability. Unlike conventional antibiotics, metal oxide nanoparticles can exert antibacterial effects through multiple simultaneous mechanisms, including membrane destabilization, reactive oxygen species (ROS) generation, oxidative stress induction, and metal ion release [12,13,14]. Such multifactorial mechanisms strongly reduce the probability of bacteria developing resistance, making inorganic nanomaterials particularly attractive for biomedical and antimicrobial applications.

Among metal oxide nanomaterials, ZnO nanoparticles have emerged as versatile multifunctional systems owing to their intrinsic antibacterial activity, low cost, chemical stability, and tunable optical and electronic properties [15,16,17]. In addition to their well-known applications in photocatalysis, sensing, and optoelectronics, ZnO nanostructures have recently attracted considerable attention for biomedical and antibacterial applications, particularly in the development of anti-infective biomaterials and surface coatings [18,19,20,21]. At the nanoscale, ZnO exhibits enhanced surface reactivity and improved interaction with bacterial membranes, leading to significant antibacterial effects against both Gram-positive and Gram-negative bacterial strains [21,22].

Recent studies demonstrated that the antibacterial response of ZnO can be significantly modulated through transition-metal doping [23,24,25], which may alter defect density, surface reactivity, ROS generation, and ion-release behavior [26,27,28]. Beyond the enhancement of antibacterial activity, doping represents an effective strategy for tailoring the physicochemical properties of ZnO by modifying its electronic structure, defect chemistry, charge carrier dynamics, and surface interactions [29]. Among the various dopants, manganese (Mn) represents a particularly interesting candidate because its ionic radius is close to that of Zn^2+^, favoring substitutional incorporation into the ZnO lattice without substantial modification of the host crystal structure. Mn is an essential trace element involved in numerous biological and enzymatic processes and exhibits relatively low toxicity at controlled concentrations [30,31]. Moreover, Mn incorporation into the ZnO lattice has been reported to influence the optical and electronic properties of ZnO, modifying defect-related states and charge transfer processes while simultaneously enhancing antibacterial performance [32].

Several synthetic approaches for the preparation of Mn-doped ZnO nanostructures have been reported in the literature, including hydrothermal methods, sol–gel synthesis, coprecipitation, combustion synthesis, and chemical vapor deposition [33,34]. Among them, wet chemical and sol–gel approaches are particularly attractive because they allow the preparation of highly dispersed nanostructures through simple, low-cost, and scalable procedures operating under mild experimental conditions [35]. These methods provide good control of the nanoparticle size, morphology, and dopant concentration by tuning the operating conditions of synthesis [36].

Besides the direct antibacterial activity against planktonic bacterial cells, increasing attention has recently been devoted to the ability of nanomaterials to interfere with bacterial virulence mechanisms and biofilm development [37,38,39]. In particular, ZnO-based nanostructures were recently reported to significantly affect bacterial adhesion and biofilm maturation [40,41,42]. Biofilm-associated infections are particularly problematic because sessile bacterial colonies exhibit enhanced tolerance toward antibiotics and disinfectants compared to free-floating bacteria [43]. In the case of *Pseudomonas aeruginosa*, virulence is strongly associated with the production of metabolites and signaling molecules, including pyoverdine and pyocyanin, which are involved in iron acquisition, oxidative stress regulation, quorum sensing, and biofilm maturation [44,45]. Therefore, the possibility of simultaneously inhibiting bacterial growth, biofilm formation, and virulence-related metabolic pathways represents a highly desirable feature for advanced antibacterial nanomaterials.

In our previous studies, we demonstrated that Mn incorporation into ZnO nanostructures significantly improves the antibacterial performance of ZnO-based systems against both Gram-positive and Gram-negative bacteria [32,46]. In particular, Mn-doped ZnO nanomaterials exhibited enhanced antibacterial activity compared to undoped ZnO, likely due to the combined contribution of modified electronic properties, ROS generation, and metal ion release mechanisms [32]. Based on these previous findings, Mn-doped ZnO nanoparticles containing different Mn nominal concentrations were synthesized through a simple, rapid, and cost-effective wet chemical approach. This approach uses Zn(II) and Mn(II) acetate salts as metal precursors in dimethyl sulfoxide (DMSO) solvent, which stabilizes the nanostructures in the fluid suspension. Their antibacterial activity was evaluated against *Staphylococcus aureus* and *Pseudomonas aeruginosa*, selected as representative Gram-positive and Gram-negative pathogens commonly involved in biomaterial-associated infections. Particular attention was focused on *P. aeruginosa*, owing to its remarkable biofilm-forming ability and the production of virulence-associated metabolites. The present work systematically investigates the influence of Mn doping concentration on both the physicochemical properties and the antibacterial performance of ZnO nanoparticles. By combining structural characterization with antibacterial, antibiofilm, and anti-virulence evaluations, it demonstrates a concentration-dependent relationship between Mn incorporation and multifunctional antibacterial activity. These findings provide valuable insights for the rational design of ZnO-based antibacterial nanomaterials used in biomedicine and biotechnology and support the development of cost-effective biomaterials for potential clinical applications.

## 2. Materials and Methods

### 2.1. Materials

Zn(II) acetate dihydrate, Mn(II) acetate tetrahydrate, dimethyl sulfoxide (DMSO, HPLC grade), and tetramethylammonium hydroxide (25 wt%) were purchased from Sigma-Aldrich (Milan, Italy) and used as received without further purification.

### 2.2. Synthesis of ZnO and Mn-Doped ZnO NPs

ZnO and Mn-doped ZnO NPs were synthesized at 23 °C by a wet-chemical coprecipitation method adapted from the procedure proposed by Panasiuk et al. [36].

For the synthesis of undoped ZnO nanoparticles, 878 mg of Zn(CH_3_COO)_2_·2H_2_O (4 mmol) were dissolved in 20 mL DMSO under constant magnetic stirring. After complete solubilization, 3 mL of tetramethylammonium hydroxide N(CH_3_)_4_OH (25 wt%) was added as a hydroxide source to induce Zn^2+^ precipitation and ZnO formation according to Equation (1).

Zn^++^ + 2OH^−^ ⇌ Zn(OH)_2_ (s) ⇌ ZnO (s) + H_2_O (1)

The reaction mixture was maintained under stirring at 23 °C for 30 min.

Mn-doped ZnO nanoparticles (Mn-ZnO NPs) were prepared through the simultaneous coprecipitation of Zn^2+^ and Mn^2+^ ions. The synthesis involves the use of Zn(CH_3_COO)_2_·2H_2_O and Mn(CH_3_COO)_2_·4H_2_O as precursors of Zn^2+^ and Mn^2+^ metal ions. Appropriate amounts of precursors (see Table 1) were dissolved in 20 mL of DMSO to obtain nominal Mn contents of 2.5, 5.0, 7.5, and 10.0 mol%, while maintaining the total metal-ion amount at 4.0 mmol and the initial total metal-ion concentration at [M^2+^] = 0.2 M (where [M^2+^] = [Zn^2+^] + [Mn^2+^]). After complete dissolution of the precursors, 3.00 mL of N(CH_3_)_4_OH (25% *w*/*w*) was added in DMSO medium under constant stirring to induce Zn^2+^ and Mn^2+^ coprecipitation and formation of the Mn-doped ZnO NPs. The reaction mixtures were stirred at 23 °C for 30 min [36,46] according to Equation (2).

xZn^++^ + (1 − x)Mn^++^ + 2OH^−^ ⇌ Zn_x_Mn_1−x_(OH)_2_ (s) ⇌ Zn_x_Mn_1−x_O (s) + H_2_O (2)

The purification was carried out by five centrifugation cycles at 5000 rpm for 10 min each. After each cycle, the supernatant was removed and the nanoparticle pellet was redispersed in DMSO. After the final purification step, each sample was resuspended in 20 mL of DMSO and sonicated for 10 min, yielding one undoped ZnO suspension and four Mn-doped ZnO suspensions with an approximate nanoparticle concentration of 16 mg/mL. The complete composition of each obtained sample is reported in Table 1.

### 2.3. UV–Vis Characterization

UV–Vis absorption spectra of the ZnO and Mn-ZnO NPs in DMSO (10 µL of the mother suspension in 3 mL of pure DMSO) suspensions were recorded in 1 cm quartz cuvettes by using a Jasco V-570 spectrophotometer (JASCO Corporation, Tokyo, Japan) operating with 2 nm slits.

### 2.4. Transmission Electron Microscopy (TEM), Energy Dispersive X-Ray (EDX) Characterization and Electron Energy Loss Spectroscopy (EELS) Characterization

TEM, STEM, EDX and EELS characterization of ZnO and Mn-ZnO nanoparticles was performed by drop-casting the nanoparticle suspensions onto gold grids coated with a thin lacey carbon film, followed by vacuum drying for 24 h.

Electron microscopy analyses were carried out using a JEOL ARM200F microscope (JEOL Ltd., Tokyo, Japan) equipped with a cold field-emission gun (CFEG) operated at an accelerating voltage of 200 kV and a probe aberration corrector, providing a spatial resolution down to 0.6 Å. Bright-field TEM imaging and scanning transmission electron microscopy (STEM) in high-angle annular dark-field (HAADF) mode were employed to investigate the morphology and microstructure of the nanoparticles.

Chemical characterization was performed by Energy Dispersive X-ray Spectroscopy (EDX) using a 100 mm^2^ silicon drift detector installed on the microscope. EDX analyses were used to verify the elemental composition of the synthesized nanomaterials and to evaluate the presence of Mn in the doped samples.

Electron Energy Loss Spectroscopy (EELS) analyses were performed in STEM mode using a GATAN GIF Quantum (Gatan Inc., Pleasanton, CA, USA) operated in dual EELS mode. Spectra were acquired from representative nanoparticle aggregates using a probe-corrected STEM configuration. Prior to edge analysis, the spectra were processed through standard background subtraction procedures. The analyses focused on the O-K, Mn- L_2,3_ and Zn-L edges. Particular attention was devoted to the Mn-L_2,3_ edge region (approximately 640–660 eV), which was used to investigate the local electronic structure and chemical environment of Mn atoms. The Mn oxidation state was assessed from both the energy position of the Mn- L_2,3_ edges and the Mn-L_3_/L_2_ intensity ratio according to established literature procedures.

Nanoparticle size analysis was performed using ImageJ software (version 1.53a). Five representative TEM/STEM micrographs were analyzed for each investigated sample. Due to the dense aggregation of nanoparticles on the TEM grids, automated image segmentation was not considered sufficiently reliable. Therefore, particle diameters were measured manually only for nanoparticles exhibiting clearly distinguishable boundaries. The particle diameter was defined as the largest apparent dimension measured between two opposite and clearly identifiable particle edges. Mean particle diameter and standard deviation were calculated from all measured nanoparticles for each sample.

### 2.5. Raman Characterization

The Raman measurements were performed by drop-casting all the NPs suspensions onto a Raman-grade CaF_2_ commercial slide and letting them dry. The CaF_2_ was used as a support substrate to avoid any spurious fluorescence contribution [47]. Raman spectra were acquired using a Horiba iHR550 spectrometer (Horiba, Kyoto, Japan) with a 600 lines/mm grating. The spectrometer is linked to an Olympus BX51 microscope equipped with a Super-Head for the selection of the 473 nm laser line (solid-state COBOLT—HÜBNER Group, Kassel, Germany). Measurements were performed by focusing 5 mW of excitation light through a 100X objective with a numerical aperture (NA) of 0.9. The backscattered signals were collected for 10 s and detected by a Peltier-cooled Syncerity CCD (Horiba). All spectra were recorded in a range between 100 and 4000 cm^−1^ to detect the whole vibrational mode. Finally, Raman spectra were processed by the software LabSpec 6 (version 6.1.180 beta) from Horiba.

### 2.6. Antibacterial Activity Evaluation

#### 2.6.1. Bacteria Strain, Media and Growth Conditions

*Staphylococcus aureus* ATCC29213 and *Pseudomonas aeruginosa* ATCC27853, purchased from the American Type Culture Collection (LGC Promochem, Milan, Italy), were cultured in tryptone soy broth (TSB, Sigma-Aldrich, Milan, Italy) and Luria–Bertani broth (LB, Sigma-Aldrich, Milan, Italy), respectively. For long-term storage, strains were maintained at −80 °C in their respective media added with 20% glycerol. At the same time, plate cultures (work plates) for each strain were prepared and stored at 4 °C to provide active bacterial cultures readily available for all antibacterial assays. The protocols used had the main goal of maintaining the vitality and genetic stability of the different bacterial species under study.

#### 2.6.2. Bacterial Growth Inhibition Activity and Minimum Inhibitory Concentration (MIC)

Bacterial growth inhibition was determined by broth microdilution assay. For each strain, semi-exponential broth culture was prepared at a final concentration of about 10^5^ bacteria/mL starting from 0.5 Mc Farland inoculum (~1.5 × 10^8^ bacteria/mL). Bacterial suspensions, dispensed in tubes, were incubated with several concentrations of Zn_x_Mn_1−x_O (600, 300, 150, 75, and 37.5 µg) NPs. Then, 150 μL from each experimental condition was distributed in 96-well plates and incubated at 37 °C for 18 h. Finally, bacterial growth was quantified by spectrophotometric reading at 540 nm (OD_540_) with a microtiter plate reader (Multiskan GO, Thermo Scientific, Waltham, MA, USA) to determine the antibacterial properties. In order to reduce the impact of NPs absorbance on the spectrophotometric reading, OD_540_ values were normalized using Equation (3) [32]

(3) $${Norm.OD}_{540}=\frac{{OD}_{540}(NPs)-{OD}_{540}(blank)}{{OD}_{540}(blank)}$$

where NPs represents Mn_x_Zn_1−x_O nanoparticles with different Zn and Mn metal ion content.

Growth reduction percentage (%*G_r_*) under each cultural condition was calculated as a percentage relative to the CTR^+^ (bacteria culture without NPs) using Equation (4)

(4) $$G_{r}(\%)=\frac{{normalized\;OD}_{540}{(CTR}^{+})-{normalized\;OD}_{490}(NPs)}{normalized\;{OD}_{490}{(CTR}^{+})}\times 100$$

MIC_90_, or Minimum Inhibitory Concentration 90%, was defined as the lowest concentration of NPs producing ≥ 90% growth inhibition.

#### 2.6.3. Biofilm Inhibition Activity and Minimum Biofilm Inhibitory Concentration (MBIC)

The ability to produce biofilm was evaluated in 96-well polystyrene microtiter plates, according to the procedure described by Coffey and Anderson [48] with some modification. Overnight cultures of *P. aeruginosa* and *S. aureus* ATCC29213 were first adjusted to a turbidity equivalent to 0.5 McFarland in the culture media with the appropriate concentration of ZnO or Mn-doped ZnO nanoparticles, selected on the basis of the bacterial growth inhibition previously determined by broth microdilution assays. Bacterial suspensions were dispensed into flat-bottom 96-well polystyrene microtiter plates together. The plates were incubated under static conditions at 37 °C for 48 h to allow for the development of mature biofilms. Following incubation, non-adherent (planktonic) bacteria were removed by washing each well three times with 300 μL of phosphate-buffered saline (PBS, pH 7.4). Each washing step consisted of filling the well, gently shaking for 5 min, and then removing the liquid. After the final wash, 200 μL of PBS and 50 μL of XTT solution (prepared according to the manufacturer’s instructions) were added to each well. The plates were incubated in the dark at 37 °C for 3 h. Subsequently, gentle agitation was applied, and absorbance was measured at 490 nm using a Perkin Elmer Wallac 1420 Victor2 Microplate Reader (Turku, Finland). Absorbance values were corrected by subtracting the background levels of the corresponding uninoculated media.

Percentage of biofilm reduction (%*B_r_*) under each cultural condition was calculated as a percentage relative to the CTR+ (bacteria culture without NPs) using Equation (5).

(5) $$B_{r}(\%)=\frac{{OD}_{490}{(CTR}^{+})-{OD}_{490}(NPs)}{{OD}_{490}{(CTR}^{+})}\times 100$$

MBIC_90_, or Minimum Biofilm Inhibitory Concentration 90%, was defined as the lowest concentration of NPs producing ≥ 90% biofilm inhibition.

#### 2.6.4. Phenazine Assay

Phenazine production from *P. aeruginosa* was quantified according to the protocol reported by Zanni et al. [49]. Specifically, 800 μL from each experimental condition were transferred into sterile microtubes and centrifuged at 6000× *g* for 10 min. Centrifugation was necessary to remove bacterial cells and avoid their interference with the spectrophotometric absorbance readings. The resulting supernatant was transferred into a 96-well plate at 200 μL per well, in triplicate for each condition, and analyzed using a Perkin Elmer Wallac 1420 Victor2 Microplate Reader (Turku, Finland). Background absorbance from fresh, uninoculated media was subtracted. Pyocyanin production was quantified by measuring absorbance at 405 nm. Measurements were performed at 48 h, corresponding to the time point at which maximum pigment production was observed. This time point was also selected because they coincide with key stages in *P. aeruginosa* biofilm development, with 48 h representing an advanced maturation phase, enabling the correlation between pigment synthesis and biofilm formation capacity under the tested conditions. Percentage of pyocyanin reduction (PYO) under each cultural condition was calculated as a percentage relative to the CTR+ (bacteria culture without Mn-ZnO NPs) using Equation (6)

(6) $$\%\;PYO=\frac{{OD}_{405}{(CTR}^{+})-{OD}_{405}(NPs)}{{OD}_{405}{(CTR}^{+})}\times 100$$

#### 2.6.5. Statistical Analysis

All results are expressed as the arithmetic mean ± standard deviation (SD) from three independent biological replicates and are reported as a percentage relative to the control condition (CTR^+^). Statistical significance was evaluated using one-way ANOVA followed by Tukey’s post hoc test for multiple comparisons (Tables S5–S9). Adjusted *p*-values < 0.05, <0.01, and <0.001 are indicated by one (*), two (**), and three (***) asterisks, respectively.

## 3. Results and Discussion

**Figure 1:**
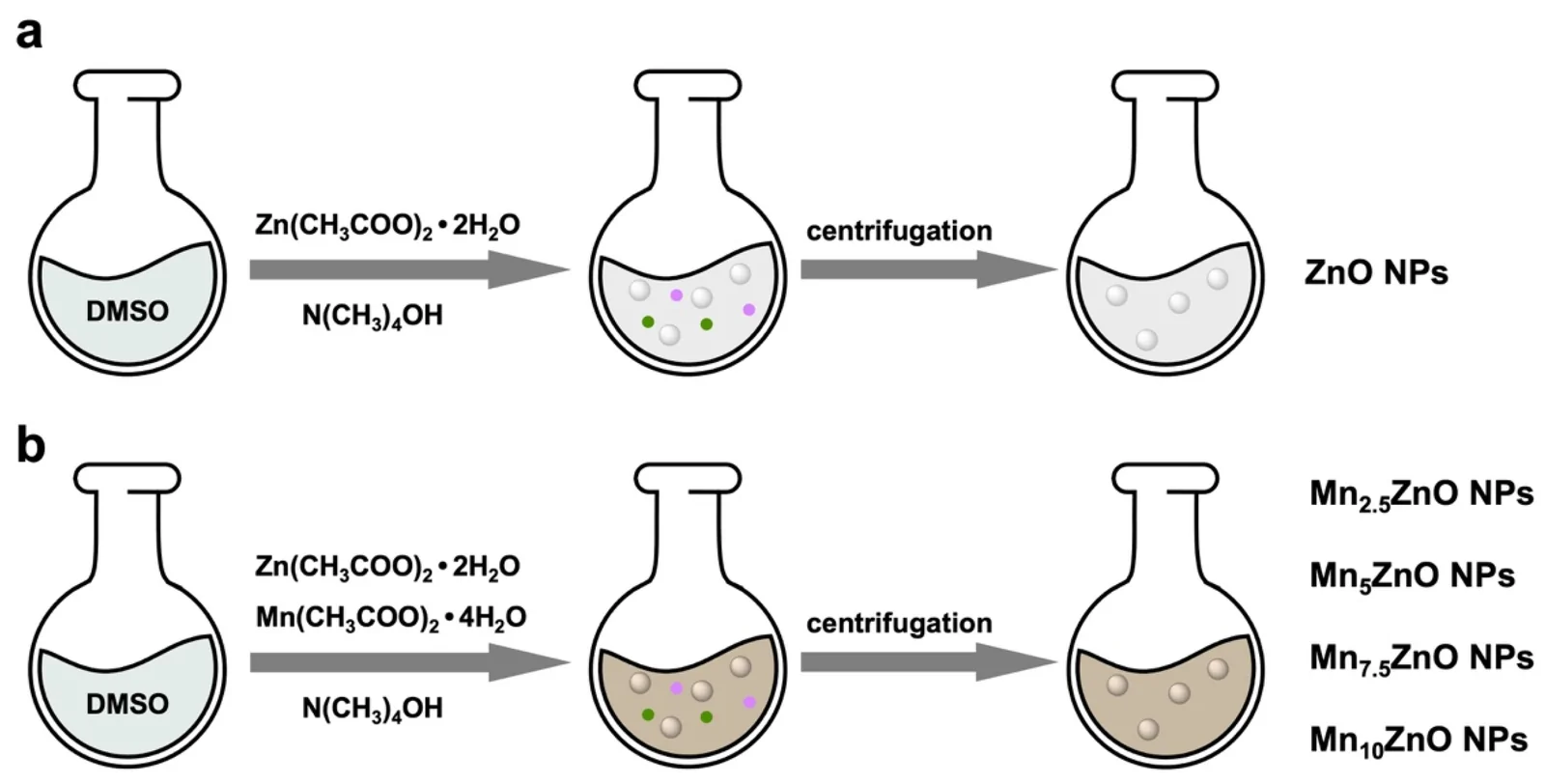
Schematic representation of the synthesis process of ZnO NPs (**a**) and Mn-doped ZnO NPs (**b**). White and brown dots represent ZnO and Mn-ZnO NPs, respectively, whereas green and magenta dots represent reaction by-products removed during the purification process.

The synthesis of ZnO and Manganese-doped ZnO (Mn-ZnO) NPs is pictured in Figure 1. Figure 1a shows the scheme for the synthesis of the undoped ZnO sample, where only Zn(CH_3_COO)_2_ was used as a source of Zn^2+^ ions in DMSO solution. Figure 1b shows the synthesis scheme for ZnO NPs doped with different amounts (%) of Mn. In this case, Zn(CH_3_COO)_2_ and Mn(CH_3_COO)_2_ are simultaneously mixed in different ratios (see paragraph 2.2 in the Section 2) to obtain a homogeneous mixture of Zn^2+^ and Mn^2+^ ions in DMSO solution. The co-precipitation of the metal ions is induced by the addition of the base N(CH_3_)_4_OH. NPs purification was performed by centrifugation, resulting in pure nanoparticles, well dispersed, and free from unreacted precursor residues in DMSO suspension. All samples synthesized by this procedure are listed in Figure 1.

**Figure 2:**
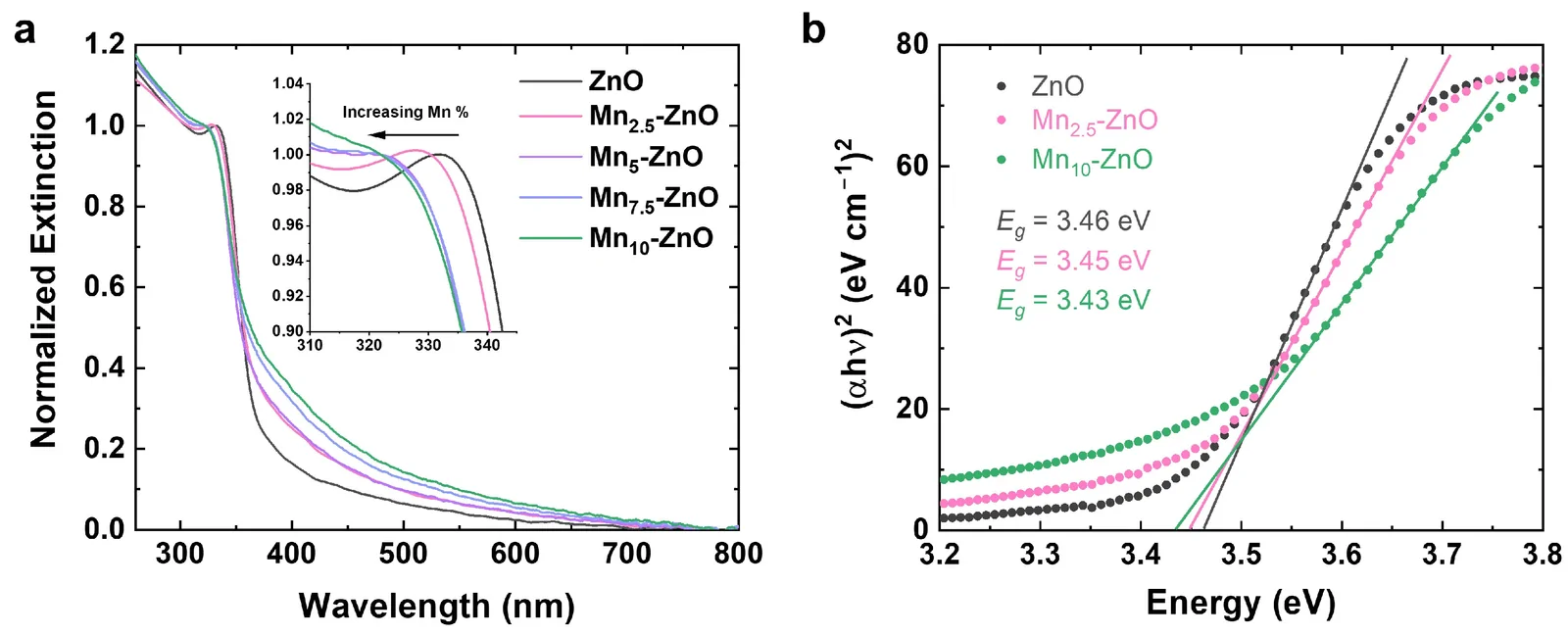
(**a**) Normalized UV–Vis extinction spectra of the synthesized ZnO and Mn-doped ZnO NPs. The inset highlights the 305–345 nm region corresponding to the ZnO matrix absorption edge. (**b**) Tauc Plot graphs used for band gap calculations of ZnO NPs (black dots), Mn_2.5_-ZnO (magenta dots), and Mn_10_-ZnO (green dots).

The normalized UV–Vis extinction spectra at RT of the NPs in DMSO suspension are shown in Figure 2a. The whole set of synthesized samples (undoped ZnO and Mn-doped ZnO) was analyzed. All the spectra show an intense light absorption at high energies, from 300 nm up to ~400 nm, and a moderate light absorption (and scattering) extending to all the visible regions. The absorption maximum observed in the ZnO NPs sample, centered at 332 nm, is associated with the electronic transitions from the valence to the conduction band [50]. In the Mn-doped NPs spectra, a blue shift in the maximum absorption edges up to ~323 nm is observed. This trend is clearly visible in the inset of Figure 2a, which reports a magnification of the spectral region between 305 nm and 345 nm. This result is consistent with the modification of the ZnO electronic structure due to the incorporation of Mn^2+^ ions [51,52]. Furthermore, increasing the Mn concentration produces an increase in the light absorption from 350 nm to low energies (up to ~600 nm) as a function of the Mn concentration. This increased absorption results in a more intense brown coloration of the Mn-ZnO suspensions compared with the white appearance of the undoped ZnO suspension and the undoped ZnO nanoparticles.

Figure 2b reports the direct band gap calculated by the Tauc plot model for three representative samples: ZnO (black dots), Mn_2.5_-ZnO (pink dots) and Mn_10_-ZnO (green dots), corresponding to the minimum and the maximum value of the studied Mn nominal concentrations. The graph shows the features in the region between 3.2 eV and 3.8 eV, highlighting the variations in the band gap profiles. For ZnO NPs, the calculated direct band gap is 3.46 eV, while for Mn_2.5_-ZnO and Mn_10_-ZnO NPs, reductions to 3.45 eV and 3.43 eV are observed, respectively. These data are in agreement with previous studies reporting that Mn incorporation reduces the band gap of doped NPs and introduces additional electronic states within the ZnO band gap [53,54].

**Figure 3:**
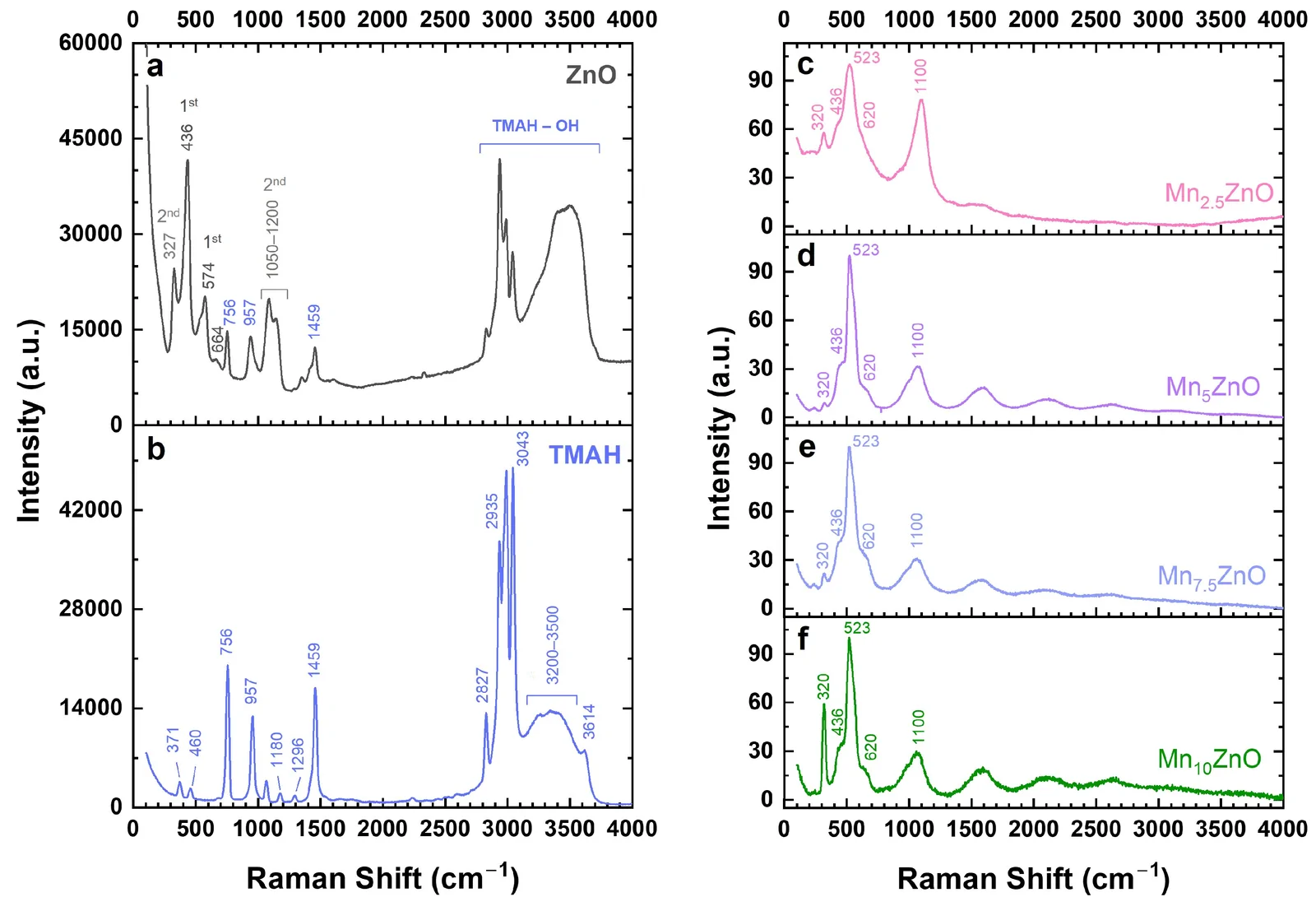
Raw Raman spectra of ZnO NPs (**a**), TMAH (**b**), Mn_2.5_-ZnO NPs (**c**), Mn_5_-ZnO NPs (**d**), Mn_7.5_-ZnO NPs (**e**), and Mn_10_-ZnO NPs (**f**) deposited onto a Raman grade CaF_2_ slide. All spectra are acquired in solid state and in the 100–4000 cm^−1^ spectral range.

The vibrational features of the synthesized ZnO and Mn-doped ZnO NPs were investigated by using Raman spectroscopy as a powerful and versatile tool widely employed for the characterization of nanostructures [55,56,57,58]. Figure 3a shows the Raman spectrum of undoped ZnO NPs, acquired in the 100–4000 cm^−1^ spectral range. Here it is possible to distinguish the transversal optical (TO) and longitudinal optical (LO) phonon components of both A_1_ and E_1_ modes of ZnO [59]. The most intense peak, centered at 436 cm^−1^, is associated with a $E_{2}^{high}$ first-order Raman mode, which involves the simultaneous vibration of two zinc atoms and two oxygen atoms [60,61]. In the range between 560 cm and ^1^ and 600 cm^−1^, the observed vibrational contributions result from the superposition of two first-order Raman modes: A_1_(LO) and E_1_(LO) centered at 574 cm^−1^ and 583 cm^−1^, respectively [59]. Due to their close frequencies, these two modes often appear convoluted into a single broad band [60].

The second-order Raman modes of ZnO are also clearly visible in the spectrum. The first signal is observed at 332 cm^−1^ and is ascribed to the combination of ${3E}_{2}^{high}-E_{2}^{low}$ modes [60,61,62]. However, this peak is centered at 327 cm^−1^ due to convolution with the CaF_2_ Raman mode (320 cm^−1^) used as a supporting slide [47]. The second peak is represented by a broad band in the 1050–1200 cm^−1^ spectral region attributed to a complex network of vibrational modes involving combinations of TO and LO components [62]. A weak Raman signal centered at 664 cm^−1^ is attributed to 2($E_{2}^{high}-E_{2}^{low}$) mode, corresponding to an opto-acoustic (TA + LO) transition with A_1_ symmetry [61,62,63]. Finally, the vibrational modes centered at 576 cm^−1^, 957 cm^−1^, and 1459 cm^−1^ are ascribed to the presence of TMAH used for the synthesis of all NPs (see Figure 3b for its reference Raman spectrum). The presence of TMAH is further confirmed by the spectral features in the high-frequency region between 2800–3700 cm^−1^, where the characteristic TMAH vibrational modes can be identified according to the classical vibrational studies reported by Anhouse et al. and Michaud et al. [64,65]. Despite purification by centrifugation, residual traces of TMAH remain detectable, suggesting that TMAH may act as a capping agent for the ZnO nanoparticles. For easier identification, all signals attributed to TMAH are marked in blue in Figure 3a. This vibrational feature is consistent with a wurtzite-type ZnO structure with 4 atoms in the hexagonal unit cell structure with C_6v_ symmetry [59,60], identified by the irreducible representation (*Γ*) reported in Equation (7).

(7) $$\Gamma =2A_{1}+2B_{1}+2E_{1}+2E_{2}$$

The incorporation of Mn into the ZnO matrix notably influences the line shape of the Raman spectra. The Mn-doped ZnO samples exhibit largely featureless spectra (Figure 3c–f) compared with the undoped one, probably due to structural and surface modifications induced by Mn doping. The first-order Raman mode at 436 cm^−1^ exhibits a drastic reduction in intensity in all doped samples. This could be due to the loss of the crystal translational symmetry caused by defects and impurities [63]. A prominent band centered at 523 cm^−1^ is observed in all Mn-doped samples, attributed to local vibration modes involving Mn atoms. Within this broad band, the polar A_1_(LO)/E_1_(LO) components are also present, exhibiting a shift toward higher frequencies [63]. The broad band observed between 600 and 700 cm^−1^ is common to all Mn-doped ZnO samples and could be associated with Mn oxide-related vibrational contributions compatible with Hausmannite-type structures (Mn^(II)^Mn^(III)^_2_O_4_) [66] incorporated within the ZnO matrix. Additionally, the broad Raman peak between 1050 cm^−1^ and 1180 cm^−1^ is present in all Mn-doped samples, in agreement with previous observations reported by Silambarasan et al. [63]. Finally, the multiple signals at high-frequency detected in samples with Mn concentrations above 2.5% (Figure 3d–f) could be associated with *n*-LO multi-phonon modes, similar to those reported in other Mn-doped ZnO systems under resonant Raman scattering (RRS) conditions [67]. An excitation wavelength of 473 nm may be enough to achieve resonance conditions, particularly in doped nanoparticles, where Mn enhances visible optical absorption. Moreover, the spectra show no detectable TMAH contributions in any of the doped nanoparticles. This suggests that Mn affects the surface chemistry of the NPs, possibly preventing TMAH adsorption. The assignment of all vibrational contributions identified in the Raman spectra shown in Figure 3 is listed in Table 2 and Table 3.

**Figure 4:**
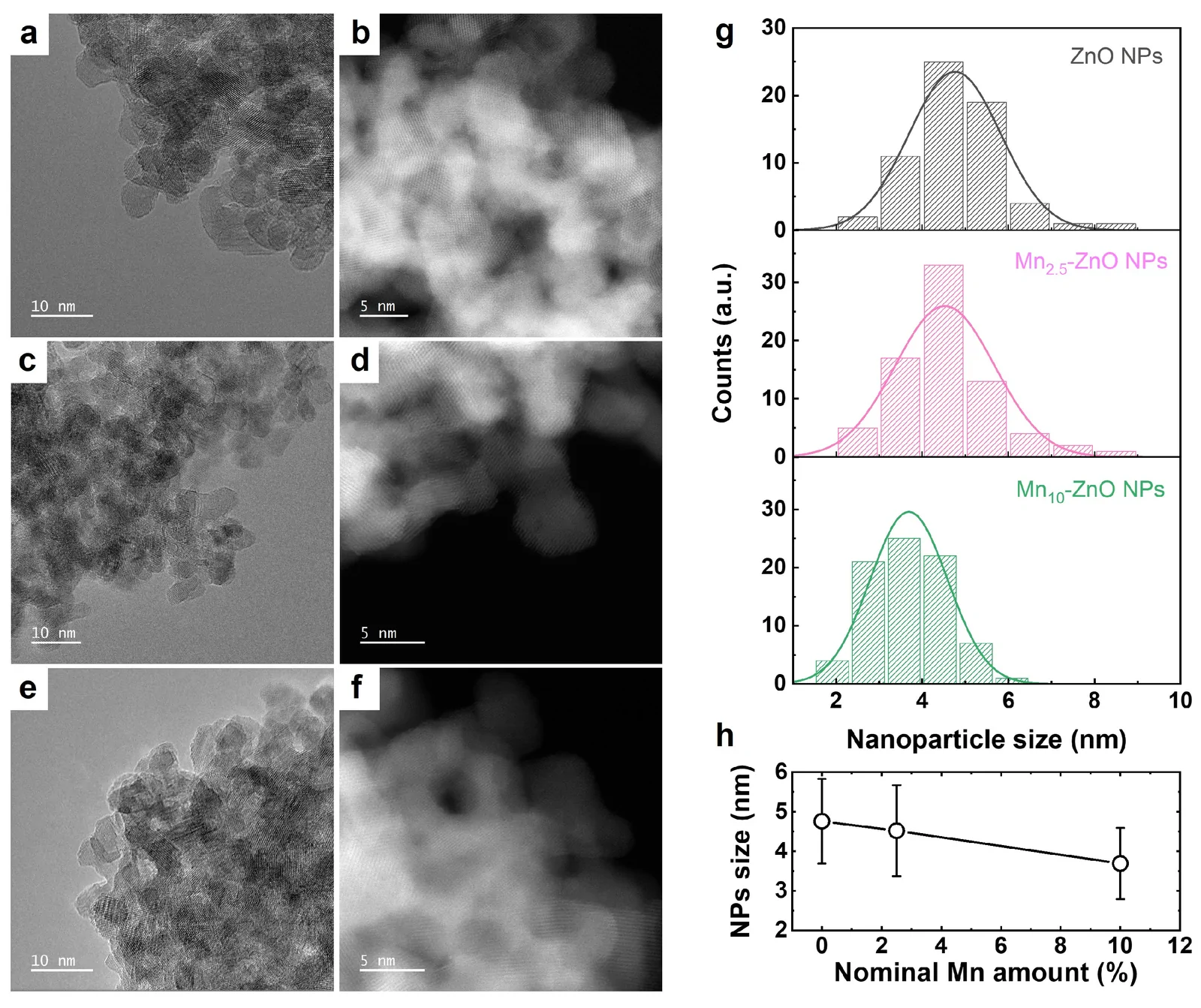
Morphological characterization of NPs by Scanning Transmission Electron Microscopy (S/TEM). Images of ZnO NPs (**a**,**b**), Mn_2.5_-ZnO NPs (**c**,**d**), and Mn_10_-ZnO NPs (**e**,**f**) acquired in bright-field (**a**,**c**,**e**) and high-angle annular dark field (**b**,**d**,**f**). (**g**) Size distribution graphs of the NPs. (**h**) Variation in the NPs mean size diameter (± standard deviation) as a function of the Mn amount.

The morphological characteristics of the NPs were investigated by scanning/transmission electron microscopy (S/TEM), as shown in Figure 4. For S/TEM analysis, representative samples corresponding to the undoped material and to the lowest and highest nominal Mn contents (Mn_2.5_-ZnO and Mn_10_-ZnO) were selected in order to provide a qualitative assessment of the morphological evolution associated with Mn incorporation. In particular, the analysis of ZnO (Figure 4a,b), Mn_2.5_-ZnO (Figure 4c,d), and Mn_10_-ZnO (Figure 4e,f) NPs was performed in both bright-field (BF) (Figure 4a,c,e) and high-angle annular dark field (HAADF) (Figure 4b,d,f) imaging. S/TEM observations revealed the formation of nearly spherical nanoparticles with a well-defined crystalline structure, as evidenced by the clearly visible lattice planes in all investigated samples. Owing to the high particle concentration and the resulting aggregation on the TEM grids, particle-size measurements were performed manually on nanoparticles with clearly distinguishable contours. The corresponding size distribution graph, shown in Figure 4g, demonstrates that this procedure produces very small ZnO NPs and Mn-doped ZnO NPs, all smaller than 10 nm. These distributions were obtained by the analysis of five bright-field images for each sample acquired at different points on the sample grid, obtaining average nanoparticle sizes of 4.76 ± 1.07 nm, 4.52 ± 1.15 nm, and 3.7 ± 0.90 nm for ZnO, Mn_2.5_-ZnO, and Mn_10_-ZnO, respectively.

Figure 4h shows the average nanoparticle size as a function of the Mn doping amount. Although a tendency toward smaller nanoparticles with increasing Mn content can be observed, the relatively large size dispersion indicates that these differences should be interpreted qualitatively rather than as statistically robust evidence of a progressive size reduction. Nevertheless, the results suggest that Mn incorporation may moderately influence the nanoparticle growth process during synthesis.

**Figure 5:**
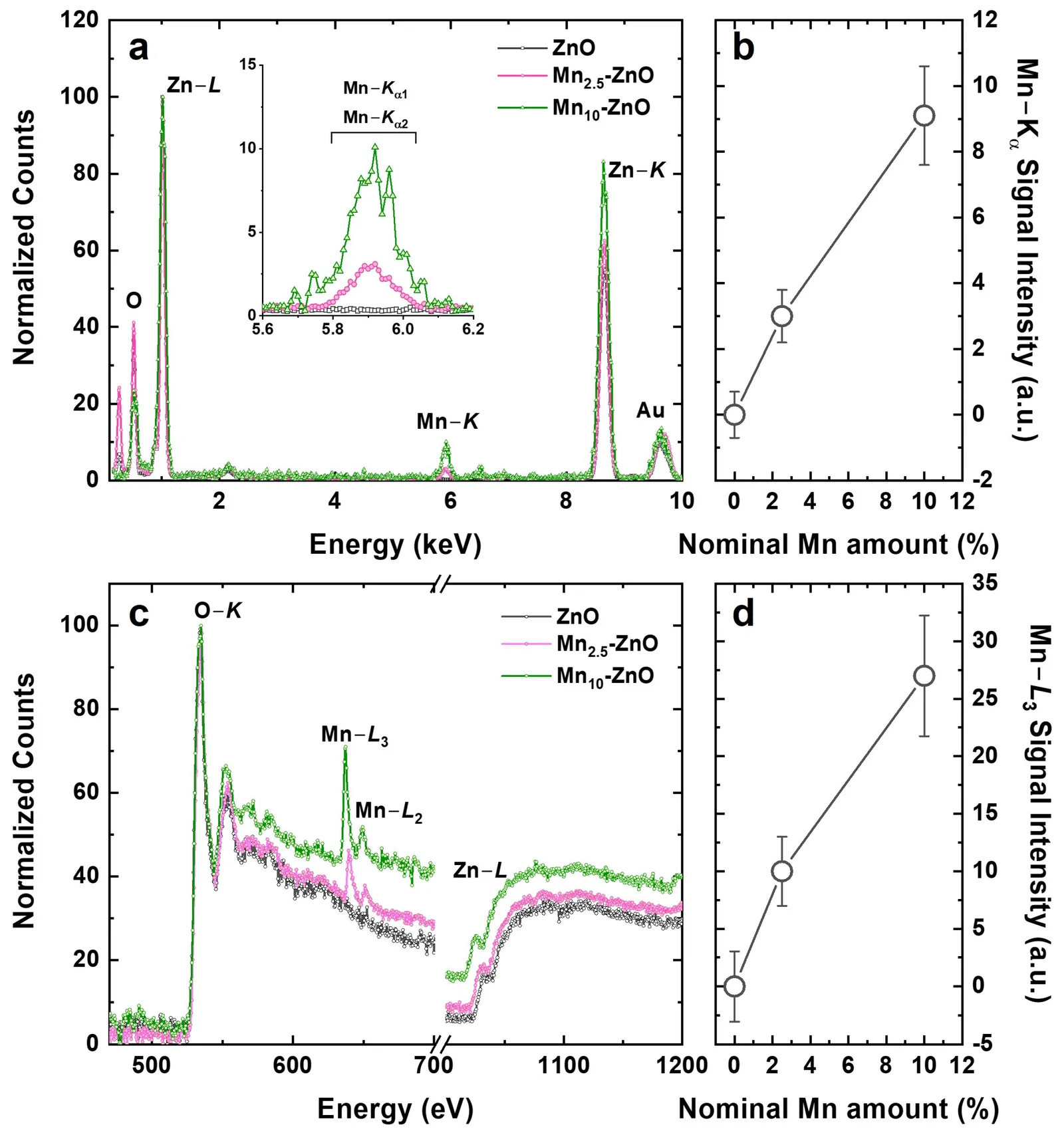
(**a**) EDX spectra of NPs. The inset shows a magnification of the 5.6–6.2 keV energy region relative to Mn-*K*_α_ transitions. (**b**) Intensity variation in the Mn-*K*_α_ band as a function of the Mn amount. (**c**) EELS spectra of NPs. The magnification of the 615–700 eV region relative to Mn-*L*_2_ and Mn-*L*_3_ emissions is shown as an inset. (**d**) Variation in intensity of the Mn-*L*_3_ bands as a function of the nominal Mn amount.

The elemental composition of pure and Mn-doped ZnO NPs was investigated by Energy Dispersive X-ray (EDX) spectroscopy (Figure 5a,b). Figure 5a shows the superposition of the EDX spectra of ZnO NPs (black line), Mn_2.5_-ZnO (magenta line) and Mn_10_-ZnO (green line). The line shape of the ZnO NPs spectrum confirms the presence of a sample composed of only zinc and oxygen, while in the Mn-doped samples the Mn signal also arises. No additional element signals were detected within the sensitivity of the EDX analysis. As highlighted in the graph, the signal at 0.52 keV is assigned to oxygen, while the signals at 1.01 keV and 8.65 keV are attributable to the *L* (*L*_α1_, *L*_α2_, *L*_β2_) and *K* (*K*_α1_, *K*_α2_) transitions of Zn [68], respectively. Finally, the signal centered at 9.6 keV is the result of the convolution of multiple contributions, with the dominant component corresponding to the gold emissions (Au-*L*_α1_ and Au-*L*_α2_) used as a sample holder grid, and weaker contributions associated with the Zn-*K*_β1_ and Zn-*K*_β2_ transitions. The inset in Figure 5a shows an enlargement of the 5.6–6.2 keV region, where the Mn-*K*_α1_ and Mn-*K*_α2_ transitions appear as a single convoluted band. As expected, increasing the Mn amount results in a concomitant increase in the Mn peak intensity from ~3 (Mn_2.5_-ZnO—magenta line) to ~9 (Mn_10_-ZnO—green line) counts, whereas the pure ZnO sample (black line) shows no detectable signal in this energy range.

This increase in Mn-K signal exhibits an approximately linear trend with increasing nominal Mn content within the investigated range (Figure 5b). Semi-quantitative EDX analyses were also performed on representative nanoparticle aggregates of the Mn_2.5_-ZnO and Mn_10_-ZnO samples. The measured Mn/(Mn + Zn) atomic ratios were 2.7 at.% and 5.9 at.%, respectively, confirming the progressive increase in Mn content with increasing nominal Mn loading. These values should be considered semi-quantitative estimates because the nanoparticles form highly aggregated assemblies on the TEM grids, leading to local variations in specimen thickness and analyzed volume that may affect the absolute X-ray intensities.

To investigate the local oxidation state and chemical environment of Mn, energy loss spectroscopy (EELS) was performed, as shown in Figure 5c. The normalized EELS edge spectra are presented for pure ZnO NPs (black line), Mn_2.5_-ZnO NPs (magenta line), and Mn_10_-ZnO NPs (green line). All spectra reveal the O-*K* edges of oxygen at 535 eV and those of zinc at 1020 eV (Zn-*L*_3_), 1145 eV (Zn-*L*_2_), and 1195 eV (Zn-*L*_1_), while in the region between 640 eV and 650 eV the Mn-*L*_3_ and Mn-*L*_2_ signals are clearly visible. Also in this case, the Mn signal intensity progressively increases with increasing Mn concentration, as shown in Figure 5d. Furthermore, the position of the *L*_3_ and *L*_2_ peaks and the *L*_3_/*L*_2_ ratios of about 3.1 and 3.8 for Mn_2.5_-ZnO NPs and Mn_10_-ZnO NPs, respectively, indicate that manganese is predominantly present as Mn^2+^ with a contribution from mixed Mn^2+^/Mn^3+^ species [69], in agreement with the Raman investigation. These results support the presence of Mn species within the ZnO-based nanostructures and provide information on their local oxidation state and chemical environment. However, EELS measurements do not provide direct information on the crystallographic phase of Mn-containing species and do not unequivocally demonstrate substitutional incorporation of Mn into the ZnO lattice. Consequently, the possible presence of minor secondary Mn-containing phases cannot be completely excluded.

The combined physicochemical and biological characterization performed in this study suggests a direct relationship between the presence of Mn-containing species in the ZnO-based nanostructures and the enhanced antibacterial performance of the resulting nanomaterials. EDX and EELS analyses confirmed Mn incorporation within the synthesized nanostructures, while UV–Vis spectroscopy revealed Mn-dependent modifications of the optical properties and a slight reduction in the band gap. These physicochemical modifications appear to be closely associated with the observed biological response.

**Figure 6:**
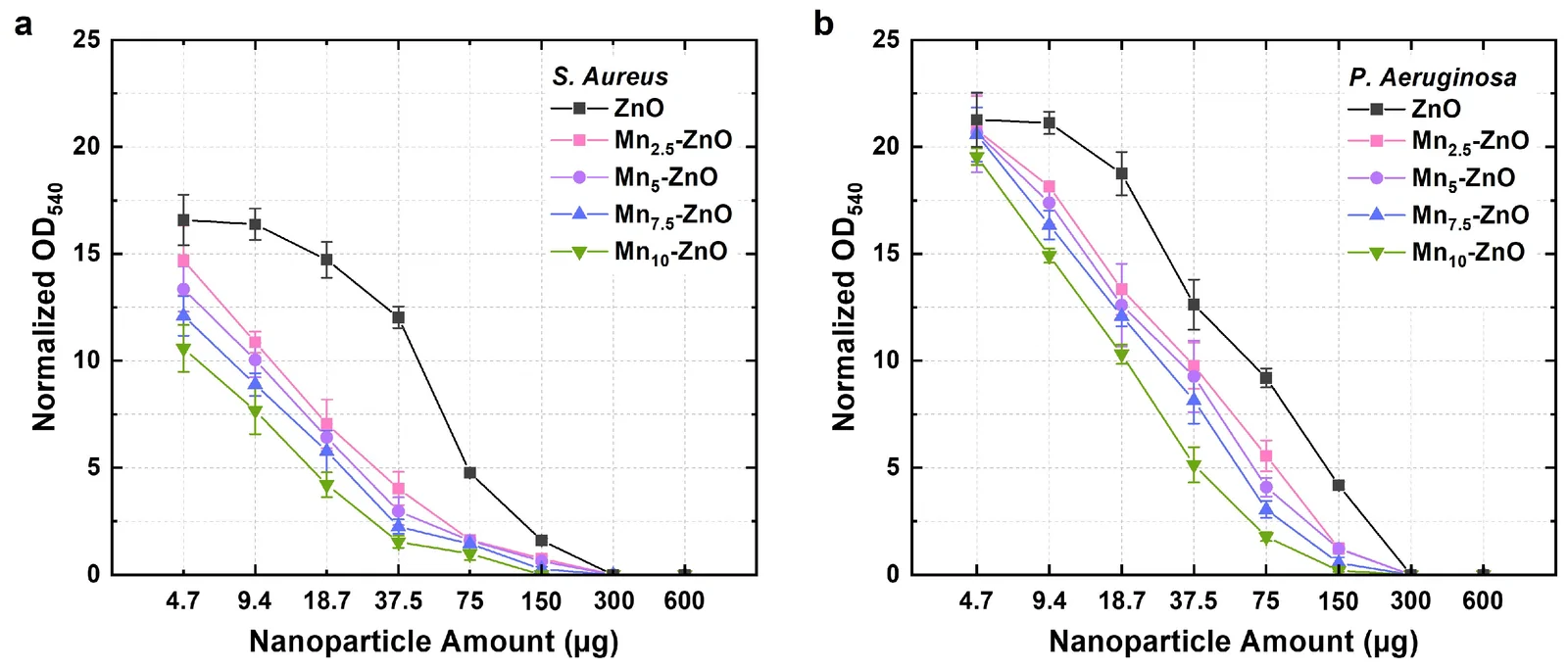
Concentration-dependent antibacterial activity of Mn-doped ZnO NPs compared with the pure ZnO NPs against *Staphylococcus aureus* (**a**) and *Pseudomonas aeruginosa* (**b**). Antibacterial activity was evaluated through normalized OD_540_ measurements after overnight incubation, corresponding to the maximum bacterial growth reached in untreated controls. Percentage of growth reduction (%Gr) and MIC_90_ values for *S. aureus* and *P. aeruginosa* are reported in Tables S1 and S2, respectively.

The antibacterial activity of pure ZnO NPs and Mn-doped NPs was evaluated against *Staphylococcus aureus* and *Pseudomonas aeruginosa* through normalized OD_540_ measurements after overnight incubation, corresponding to the maximum bacterial growth reached in untreated controls (Figure 6). Percentage of growth reduction (%*G_r_*) and MIC_90_ values are summarized in Tables S1 and S2 for *S. aureus* and *P. aeruginosa*, respectively.

In both bacterial species, all formulations induced a clear concentration-dependent reduction in bacterial growth, confirming the antibacterial potential of both pure and Mn-doped ZnO nanostructures. For *S. aureus*, increasing nanoparticle concentrations progressively reduced bacterial growth for all tested formulations. Pure ZnO displayed the weakest antibacterial activity, reaching MIC_90_ only at 150 μg/mL (Table S1), whereas Mn-doped nanoparticles showed progressively improved antibacterial effects as Mn concentration increased. In particular, Mn_2.5_-ZnO, Mn_5_-ZnO, and Mn_7.5_-ZnO reached MIC_90_ at 75 μg/mL, while Mn_10_-ZnO achieved MIC_90_ already at 37.5 μg/mL, corresponding to 90.8% growth reduction (Table S1). A similar concentration-dependent inhibitory profile was observed against *P. aeruginosa*, although this Gram-negative species appeared overall less susceptible than *S. aureus*. Pure ZnO again showed the lowest antibacterial activity, failing to reach MIC_90_ until 300 μg/mL, whereas Mn-doped formulations displayed progressively enhanced inhibition as Mn concentration increased. Mn_2.5_-ZnO, Mn_5_-ZnO, and Mn_7.5_-ZnO reached MIC_90_ at 150 μg/mL, while Mn_10_-ZnO achieved MIC_90_ at 75 μg/mL (Table S2).

Notably, the differences among formulations became more evident at intermediate concentrations, where Mn-doped nanoparticles consistently produced higher growth reduction percentages compared to pure ZnO in both bacterial species. This effect was particularly relevant for *P. aeruginosa*, the intrinsically less susceptible species, and led to a reduction in the MIC_90_ by one dilution factor in both *S. aureus* and *P. aeruginosa* compared to pure ZnO. Specifically, the Mn_10_-ZnO NPs reduced the MIC_90_ from 150 μg/mL to 37.5 μg/mL in *S. aureus* and from 300 μg/mL to 75 μg/mL in *P. aeruginosa*. This enhanced activity correlates with the Mn-induced modifications observed in the physicochemical characterization, including reduced particle size and altered electronic properties.

The overall higher susceptibility observed for *S. aureus* compared to *P. aeruginosa* is consistent with the structural differences between Gram-positive and Gram-negative bacteria. The exposed peptidoglycan layer of *S. aureus* likely facilitates nanoparticle interaction with the bacterial envelope, resulting in higher sensitivity to ZnO-based nanomaterials [70]. Conversely, the lower susceptibility of *P. aeruginosa* is likely associated with intrinsic resistance mechanisms characteristic of Gram-negative bacteria, including the presence of an outer membrane enriched in lipopolysaccharides and efficient efflux systems that limit nanoparticle penetration and ionic diffusion. Nevertheless, Mn incorporation progressively enhanced the antibacterial activity against both bacterial species, with the Mn_10_-ZnO NPs producing the strongest effect. Similar observations have previously been reported for Mn-doped ZnO nanomaterials, where Mn incorporation was associated with enhanced ROS-mediated antibacterial activity due to band-gap reduction and increased surface redox reactivity [26]. Although antibacterial activity remained strongly concentration-dependent, Mn incorporation clearly improved the antibacterial performance of ZnO nanoparticles against both Gram-positive and Gram-negative pathogens, with a more pronounced impact on the intrinsically less susceptible *P. aeruginosa*.

**Figure 7:**
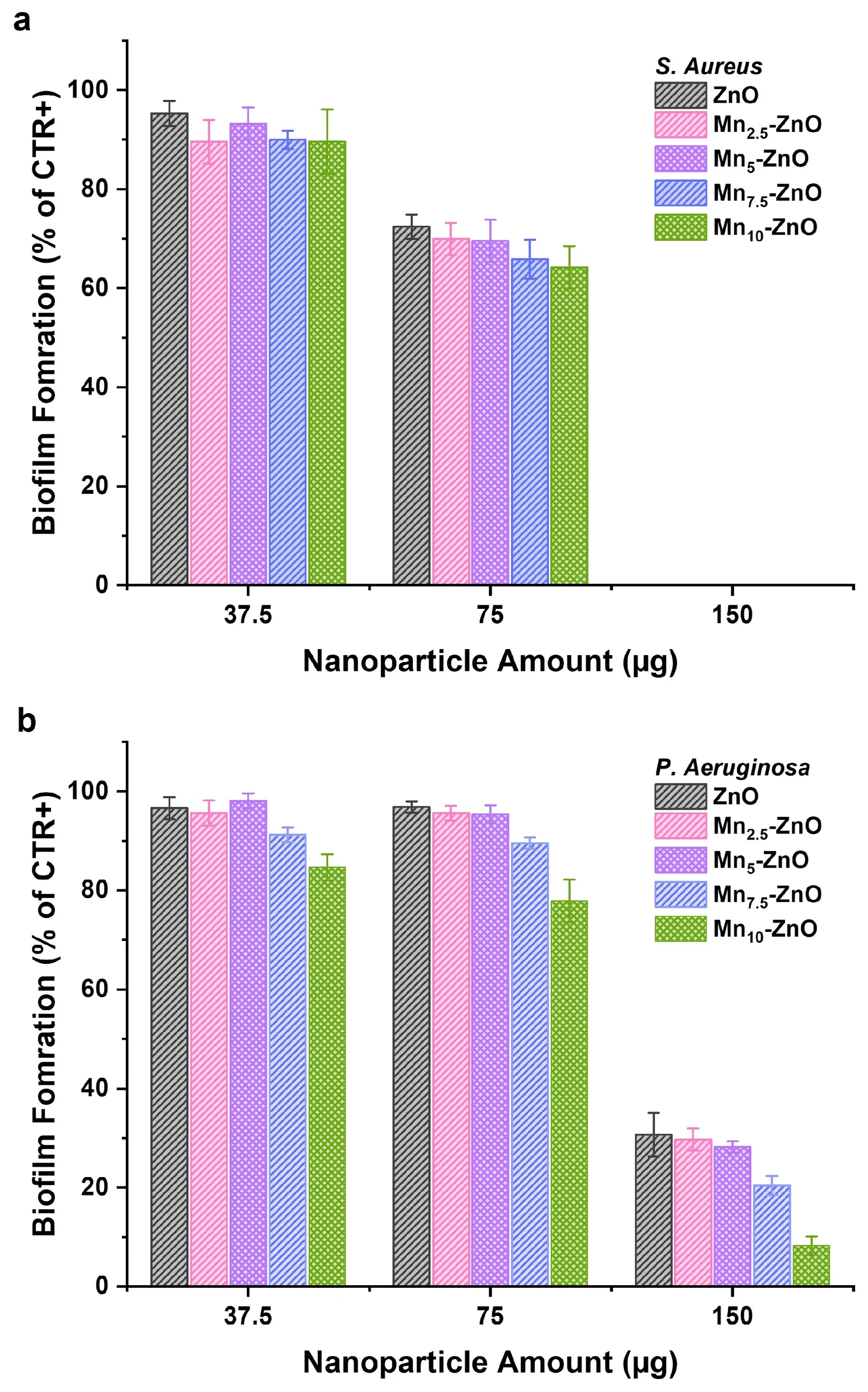
Concentration-dependent biofilm inhibition activity of Mn-doped ZnO NPs compared with the pure ZnO NPs against *Staphylococcus aureus* (**a**) and *Pseudomonas aeruginosa* (**b**). Biofilm inhibition activity was assessed by XTT-based quantification of the metabolic activity of mature biofilms formed after 48 h of incubation under static conditions. The percentage of biofilm reduction (%*B_r_*) and the corresponding MBIC_90_ values for *S. aureus* and *P. aeruginosa* are reported in Tables S3 and S4, respectively. Statistical significance was evaluated using one-way ANOVA followed by Tukey’s post hoc test for multiple comparisons (Tables S5–S9).

The ability of the nanostructures to impair biofilm formation was also evaluated (Figure 7). In *S. aureus* (Figure 7a), biofilm inhibition generally mirrored the antibacterial activity observed for planktonic cells. All Mn-doped ZnO NPs induced a concentration-dependent reduction in biofilm metabolic activity, reaching MBIC_90_ at 150 μg/mL regardless of Mn content (Table S3). Although Mn incorporation did not further reduce the MBIC_90_ value, a progressive increase in biofilm reduction was observed at sub-inhibitory concentrations, with %*B_r_* values increasing from 27.6% for pure ZnO to 35.8% for Mn_10_-ZnO NPs at 75 μg/mL. However, Tukey’s post hoc analysis revealed no statistically significant differences among the various nanoparticle formulations at either 37.5 or 75 μg/mL (Tables S5 and S6), indicating that Mn doping only marginally influenced antibiofilm activity against this highly susceptible Gram-positive species. These findings are consistent with the greater sensitivity of *S. aureus* to ZnO-based nanomaterials, likely due to the absence of an outer membrane and the resulting increased exposure to Zn^2+^-mediated oxidative stress.

A markedly different pattern was observed for *P. aeruginosa* (Figure 7b). While pure ZnO nanoparticles achieved MBIC_90_ only at 300 μg/mL, the Mn_10_-ZnO NPs reduced the MBIC90 to 150 μg/mL, corresponding to a two-fold improvement in antibiofilm efficacy (Table S4). Moreover, biofilm reduction progressively increased with Mn content across all tested concentrations. At 37.5 μg/mL, %*B_r_* increased from 3.4% for pure ZnO to 15.3% for Mn_10_-ZnO, whereas at 75 μg/mL the corresponding values increased from 3.2% to 22.1%. This trend became even more evident at 150 μg/mL, where Mn_10_-ZnO achieved 91.7% biofilm inhibition compared with 69.3% for pure ZnO. Importantly, these differences were supported by statistical analysis. Tukey’s multiple-comparison test demonstrated significant improvements for Mn_7.5_-ZnO and Mn_10_-ZnO compared with pure ZnO at both 75 and 150 μg/mL, with the strongest significance observed for the 10% Mn-doped formulation (Tables S7–S9). These results indicate that Mn incorporation substantially enhances the antibiofilm activity of ZnO nanoparticles against *P. aeruginosa*, a species characterized by a highly structured extracellular polymeric matrix and intrinsic resistance mechanisms. The stronger antibiofilm effect observed against *P. aeruginosa* closely paralleled the progressive increase in Mn content, suggesting a direct relationship between doping level and biological performance.

The comparison between the two bacterial species suggests that Mn doping exerts a limited effect on biofilm inhibition in *S. aureus*, where pure ZnO is already highly effective, whereas its contribution becomes considerably more relevant in *P. aeruginosa*. Indeed, all formulations reached the same MBIC_90_ value against *S. aureus*, and no significant differences among nanoparticle compositions were detected by Tukey’s post hoc analysis, despite a modest increase in biofilm reduction percentages with increasing Mn content. In contrast, the antibiofilm activity against *P. aeruginosa* was strongly influenced by Mn incorporation, resulting in a progressive enhancement of biofilm inhibition and a two-fold reduction in the MBIC_90_ value for the Mn_10_-ZnO NPs. The greater impact observed in *P. aeruginosa* likely reflects the higher structural complexity of Gram-negative biofilms, which are characterized by a dense extracellular polymeric substance (EPS) matrix rich in polysaccharides, extracellular DNA, proteins, and redox-active metabolites that contribute to antimicrobial tolerance [71,72].

The enhanced performance of Mn-doped nanoparticles may be associated with several complementary mechanisms. First, Mn incorporation has been reported to modify the electronic structure of ZnO, promoting charge separation and increasing reactive oxygen species (ROS) generation through enhanced surface redox activity [73]. Increased ROS production can damage cellular membranes, proteins, nucleic acids, and components of the biofilm matrix itself, thereby weakening biofilm integrity and reducing bacterial viability. In addition, Mn doping has been shown to increase the density of oxygen vacancies and surface defects in ZnO nanostructures, properties that are frequently associated with improved antimicrobial and antibiofilm performance. Such modifications may facilitate nanoparticle interactions with EPS components and promote deeper penetration into the biofilm architecture, allowing oxidative stress to reach bacterial cells embedded within the mature biofilm.

The stronger effect observed against *P. aeruginosa* may also be related to the reported ability of metal oxide nanoparticles to interfere with quorum sensing-regulated processes involved in biofilm maturation and maintenance. Although the present study did not directly investigate quorum sensing pathways or oxidative stress-related mechanisms, the enhanced antibiofilm activity observed for Mn-doped formulations, together with the marked reduction in pyocyanin production, suggests that these nanomaterials may exert a multifactorial mode of action extending beyond simple growth inhibition. The antimicrobial activity of ZnO-based nanomaterials is generally attributed to ROS generation and Zn^2+^ release, which can promote oxidative stress and membrane damage in bacterial cells and may contribute to the disruption of biofilm development and virulence-associated phenotypes [74]. Recent studies have demonstrated that Mn-doped ZnO nanostructures exhibit enhanced antimicrobial activity compared with undoped ZnO, an effect that has been associated with increased surface reactivity, ROS generation, and improved interactions with bacterial cells. These properties may also contribute to the superior antibiofilm performance observed in the present study [26]. Furthermore, the concomitant reduction in pyocyanin production observed in the present study provides additional support for the hypothesis that Mn-doped ZnO nanoparticles may affect virulence-associated phenotypes beyond their direct antibacterial activity. Therefore, although further investigations are required to clarify the molecular mechanisms involved, the combined inhibition of biofilm formation and pyocyanin production observed in the present study supports the hypothesis that Mn doping may interfere with multiple bacterial physiological processes associated with pathogenicity and virulence expression [28]. These findings support the role of Mn as a co-adjuvant element capable of potentiating the biological activity of ZnO nanoparticles, particularly against intrinsically less susceptible Gram-negative biofilms, where overcoming matrix-associated tolerance represents a major challenge for conventional antimicrobial strategies.

**Figure 8:**
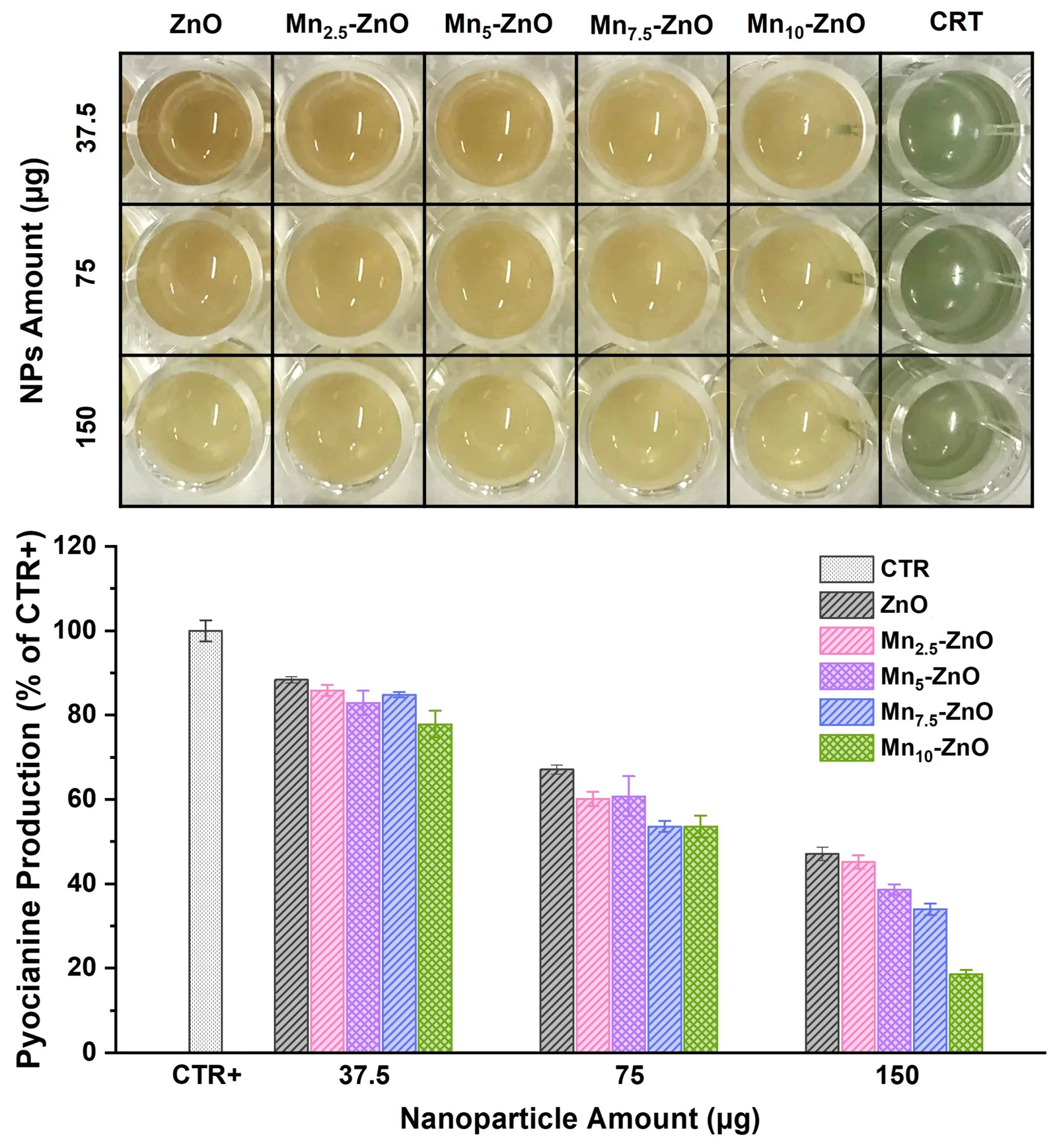
Concentration-dependent pyocyanin inhibition of Mn-doped ZnO NPs compared with the pure ZnO NPs against *Pseudomonas aeruginosa***.** Pyocyanin inhibition activity was assessed by OD_405_ after 48 h of incubation under static conditions. Statistical significance was evaluated using one-way ANOVA followed by Tukey’s post hoc test for multiple comparisons (Tables S10–S12).

The effect of bare ZnO NPs alone and Mn-doped ZnO NPs on pyocyanin production by *P. aeruginosa* was also evaluated after 48 h of incubation under static conditions (Figure 8). Pyocyanin inhibition was quantified by measuring OD_405_, and statistical significance was assessed by one-way ANOVA followed by Tukey’s multiple-comparison test (Tables S10–S12).

All nanoparticle formulations significantly reduced pyocyanin production compared with the untreated control, indicating that ZnO-based nanomaterials exert not only antibacterial and antibiofilm effects but also interfere with the production of an important virulence determinant of *P. aeruginosa*. A clear concentration-dependent inhibition of pyocyanin production was observed for all tested formulations. However, unlike the antibacterial assays, where the effect of Mn doping became more evident only at intermediate nanoparticle concentrations, pyocyanin inhibition showed a pronounced dependence on Mn content across the entire concentration range. Interestingly, pyocyanin inhibition exhibited the clearest Mn-dependent trend among all investigated biological parameters. At 37.5 μg/mL, all formulations significantly reduced pyocyanin production relative to the untreated control, but the strongest inhibition was observed for Mn_10_-ZnO, which was also significantly more effective than pure ZnO (Table S12). This trend became progressively more evident at 75 and 150 μg/mL, where increasing Mn content was associated with increasingly stronger inhibition of pyocyanin synthesis. Notably, at 150 μg/mL, significant differences were detected not only between pure ZnO and Mn_10_-ZnO, but also between Mn_5_-ZnO and Mn_7.5_-ZnO, demonstrating a progressive Mn-dependent enhancement of antivirulence activity (Table S10).

The pronounced reduction in pyocyanin production is particularly relevant because pyocyanin is one of the major virulence factors produced by *P. aeruginosa*. Beyond its role as a phenazine pigment, pyocyanin contributes to oxidative stress induction, epithelial damage, immune modulation, biofilm maturation, and persistence during chronic infections [75]. Consequently, inhibition of pyocyanin production may substantially attenuate bacterial pathogenicity even in situations where complete bacterial eradication is not achieved. The observation that pyocyanin inhibition increased progressively with Mn incorporation suggests that Mn-doped ZnO NPs affect biological processes beyond simple growth inhibition. Interestingly, the magnitude of the Mn-dependent effect observed for pyocyanin production was greater than that observed for planktonic growth inhibition and even biofilm suppression. While Mn doping reduced MIC_90_ and MBIC_90_ values by one and two dilution factors, respectively, the antivirulence response displayed a more gradual and statistically supported dose–response relationship across the entire range of Mn concentrations. This finding suggests that Mn incorporation may preferentially enhance mechanisms involved in the regulation of virulence-associated pathways. Although the molecular basis of this effect was not investigated in the present study, previous studies have shown that metal oxide nanoparticles can interfere with redox-sensitive regulatory networks and quorum sensing-dependent phenotypes in *P. aeruginosa*, ultimately reducing pyocyanin biosynthesis and other virulence-associated traits [76].

The strong correlation observed between enhanced antibiofilm activity and reduced pyocyanin production is particularly noteworthy. Pyocyanin has been reported to contribute to biofilm development through the promotion of extracellular DNA release and stabilization of the biofilm matrix [77]. Therefore, the greater antibiofilm activity observed for Mn-doped NPs, especially Mn_10_-ZnO, may be partially associated with their ability to suppress pyocyanin production. The fact that the same formulation produced the lowest MBIC_90_ value and the strongest inhibition of pyocyanin synthesis supports the hypothesis that Mn incorporation potentiates the multitarget activity of ZnO NPs, simultaneously affecting bacterial growth, biofilm formation, and virulence expression. These findings indicate that Mn doping substantially enhances the antivirulence properties of ZnO nanoparticles against *P. aeruginosa*. While all formulations reduced pyocyanin production, the progressive improvement observed with increasing Mn content, together with the highly significant differences detected by Tukey’s analysis, highlights the importance of Mn as a functional co-dopant capable of amplifying the biological activity of ZnO NPs. The ability to simultaneously inhibit bacterial growth, biofilm formation, and pyocyanin production makes Mn-doped ZnO nanoparticles particularly attractive candidates for the development of multifunctional antimicrobial strategies against persistent *P. aeruginosa* infections.

## 4. Conclusions

Mn-doped ZnO nanoparticles containing different nominal Mn concentrations were successfully synthesized through a simple wet-chemical approach. The combined UV–Vis, Raman, TEM, EDX, and EELS characterization confirmed the presence of Mn within the ZnO-based nanostructures and revealed Mn-dependent modifications of their physicochemical properties. In particular, EELS measurements indicated that Mn is predominantly present as Mn^2+^ with a minor contribution from mixed valence Mn^2+^/Mn^3+^ species.

The physicochemical modifications induced by Mn doping were followed by a progressive enhancement of biological activity, including improved antibacterial activity against both *S. aureus* and *P. aeruginosa*. The sample with the highest Mn concentration (Mn_10_-ZnO) showed the highest efficacy, reducing the MIC_90_ from 150 to 37.5 μg/mL against *S. aureus* and from 300 to 75 μg/mL against *P. aeruginosa*. The same sample also exhibited the strongest antibiofilm activity and, specifically for *P. aeruginosa*, the greatest suppression of pyocyanin production, highlighting the beneficial effect of Mn doping on both antibacterial and anti-virulence performance. These findings demonstrate that controlling Mn incorporation represents an effective strategy for engineering multifunctional ZnO nanomaterials with tunable physicochemical properties and enhanced antimicrobial activity. These findings establish a concentration-dependent relationship between Mn content, physicochemical properties, and biological activity. Mn-containing ZnO NPs therefore represent attractive building blocks for the development of anti-infective biomaterials. Their incorporation into coatings or functional surfaces could provide a strategy to limit bacterial colonization, biofilm development, and virulence at material–tissue interfaces, with potential applications in implantable medical devices and other biomedical systems. Further studies in more complex models will be required to assess their potential for practical biomedical applications.

## Figures and Tables

**Table 1:** Composition of the reaction mixtures used for the synthesis of ZnO and Mn-doped ZnO nanoparticles.

Sample Name	Zn(CH_3_COO)_2_·2H_2_O (mg)	[Zn^2+^] in DMSO (M)	Mn(CH_3_COO)_2_·4H_2_O (mg)	[Mn^2+^] in DMSO (M)	Nominal Mn/Zn % Ratio
ZnO	878.0	0.200	–	–	0/100
Mn_2.5_-ZnO	856.0	0.195	24.5	0.005	2.5/97.5
Mn_5_-ZnO	834.1	0.190	49.0	0.010	5/95
Mn_7.5_-ZnO	812.2	0.185	73.5	0.015	7.5/92.5
Mn_10_-ZnO	790.2	0.180	98.0	0.020	10/90

The total metal ion amount was maintained at 4.00 mmol in all reaction mixtures, corresponding to an initial total metal ion concentration, [Zn^2+^] + [Mn^2+^], of 0.200 M in 20 mL of DMSO.

**Table 2:** Assignment of all vibrational modes observed in Raman spectra of pure ZnO and Mn-doped NPs samples.

Frequency (cm^−1^)	Assignment	Reference
327	$E_{2}^{high}-E_{2}^{low}$, CaF_2_	[47,60,61,62]
436	$E_{2}^{high}$	[60,62,63]
523	Mn-doping	[63]
574	A_1_(LO)	[62]
620	Mn^(II)^Mn^(III)^_2_O_4_	[63,66]
664	2($E_{2}^{high}-E_{2}^{low}$)	[62]
1050–1200	TO + LO	[62]

**Table 3:** Assignment of vibrational modes observed in the Raman spectrum of the tetramethylammonium hydroxide (TMAH).

Frequency (cm^−1^)	Assignment	Reference
371	CN^+^ bending	[64,65]
460	CN^+^ bending	[64,65]
756	CN^+^ symmetric stretching	[64,65]
957	CN^+^ stretching	[64,65]
1180	CH_3_ rocking	[64,65]
1296	CH_3_ rocking	[64,65]
1459	CH_3_ asymmetric bending	[64,65]
2827	CH_3_ symmetric stretching	[64]
2935	CH_3_ symmetric stretching	[64]
2970	CH_3_ symmetric stretching	[64]
2990	CH_3_ symmetric stretching	[64]
3043	CH_3_ symmetric stretching	[64]
3200–3500	OH stretching in H_2_O	[58,65]
3614	OH^−^ stretching	[65]

## Data Availability

The original contributions presented in this study are included in the article/Supplementary Material. Further inquiries can be directed to the corresponding authors.

## Supplementary Materials

The following supporting information can be downloaded at: https://www.mdpi.com/article/10.3390/nano16161019/s1, Table S1: *S. aureus* percentage of growth reduction (%G_r_) and MIC_90_ values of ZnO nanoparticles alone and doped with 2.5–10% Mn; Table S2: *P. aeruginosa* percentage of growth reduction (%G_r_) and MIC_90_ values of ZnO nanoparticles alone and doped with 2.5–10% Mn; Table S3: *S. aureus* percentage of biofilm reduction (%B_r_) and MBIC_90_ values of ZnO nanoparticles alone and doped with 2.5–10% Mn; Table S4: *P. aeruginosa* percentage of biofilm reduction (%B_r_) and MBIC_90_ values of ZnO nanoparticles alone and doped with 2.5–10% Mn; Table S5: Tukey’s multiple-comparison analysis of *S. aureus* biofilm metabolic activity (XTT assay, OD_490_) following treatment with 75 μg/mL of pure ZnO nanoparticles or ZnO nanoparticles doped with 2.5–10% Mn; Table S6: Tukey’s multiple-comparison analysis of *S. aureus* biofilm metabolic activity (XTT assay, OD_490_) following treatment with 37.5 μg/mL of pure ZnO nanoparticles or ZnO nanoparticles doped with 2.5–10% Mn; Table S7: Tukey’s multiple-comparison analysis of *Pseudomonas aeruginosa* biofilm metabolic activity (XTT assay, OD_490_) following treatment with 150 μg/mL of pure ZnO nanoparticles or ZnO nanoparticles doped with 2.5–10% Mn; Table S8: Tukey’s multiple-comparison analysis of *Pseudomonas aeruginosa* biofilm metabolic activity (XTT assay, OD_490_) following treatment with 75 μg/mL of pure ZnO nanoparticles or ZnO nanoparticles doped with 2.5–10% Mn; Table S9: Tukey’s multiple-comparison analysis of *Pseudomonas aeruginosa* biofilm metabolic activity (XTT assay, OD_490_) following treatment with 37.5 μg/mL of pure ZnO nanoparticles or ZnO nanoparticles doped with 2.5–10% Mn; Table S10: Tukey’s multiple-comparison analysis of *Pseudomonas aeruginosa* pyocyanin production (OD_405_) following treatment with 150 μg/mL of pure ZnO nanoparticles or ZnO nanoparticles doped with 2.5–10% Mn; Table S11: Tukey’s multiple-comparison analysis of *Pseudomonas aeruginosa* pyocyanin production (OD_405_) following treatment with 75 μg/mL of pure ZnO nanoparticles or ZnO nanoparticles doped with 2.5–10% Mn; Table S12: Tukey’s multiple-comparison analysis of *Pseudomonas aeruginosa* pyocyanin production (OD_405_) following treatment with 37.5 μg/mL of pure ZnO nanoparticles or ZnO nanoparticles doped with 2.5–10% Mn.
