# Supplementary material for: Mn Doping Enhances the Antibacterial, Antibiofilm and Anti-Virulence Activity of ZnO Nanoparticles
Source: Nanomaterials (Basel). 2026 Aug 18;16(16):1019. doi: 10.3390/nano16161019 (PMC13517083; doi:10.3390/nano16161019)
Supplement: Supplementary file 1 [file nanomaterials-16-01019-s001.zip › nanomaterials-4470519-supplementary.pdf]

nanomaterials

Supplementary Information

Mn Doping Enhances the Antibacterial, Antibiofilm and Anti-Virulence Activity of ZnO Nanoparticles

Dario Morganti [1,2,+,*], Domenico Franco [2,+,*], Giuseppe Nicotra [3], Elena Spagnoli [1], Stefano Zampolli [1], Vittorio Morandi [1] and Sabrina Conoci [1,4]

1 National Research Council, Institute for Nanostructured Materials (CNR-ISMN), Bologna Section, Via Piero Gobetti 101, I-40129, Bologna (BO), Italy
2 Department of Chemical, Biological, Pharmaceutical and Environmental Sciences, University of Messina, Viale F. Stagno d'Alcontres 31, I-98166 Messina (ME), Italy
3 National Research Council, Institute for Microelectronics and Microsystems, (CNR-IMM), Catania (CT), Italy
4 Department of Chemistry G. Ciamician, University of Bologna, Via Piero Gobetti 85, I-40129, Bologna (BO), Italy

+ These authors contributed equally to this work
* Correspondence: dario.morganti@cnr.it (D.M.); domenico.franco@unime.it (D.F.)

Table S1: *S. aureus* percentage of growth reduction (%Gr) and MIC_{90} values of ZnO nanoparticles alone and doped with 2.5–10% MnO

	**4.7 µg**	**9.4 µg**	**18.8 µg**	**37.5 µg**	**75 µg**	**150 µg**	**300 µg**	**600 µg**
**$Zn_{100\%}$**	1.1%	2.3%	12.2%	28.3%	71.6%	**90.4%**	100%	100%
**$Zn_{97.5\%}Mn_{2.5\%}$**	12.4%	35.2%	57.9%	76%	***90.3%**	95.4%	100%	100%
**$Zn_{95\%}Mn_{5\%}$**	20.4%	40.1%	61.7%	82.3%	***90.5%**	96.2%	100%	100%
**$Zn_{92.5\%}Mn_{7.5\%}$**	27.9%	46.9%	65.6%	86.6%	***91.4%**	98.4%	100%	100%
**$Zn_{90\%}Mn_{10\%}$**	36.9%	54.2%	74.9%	***90.8%**	94.1%	100%	100%	100%

* Inhibition values considered for the determination of the Minimum Inhibitory Concentration 90% (MIC_{90})

Table S2: *P. aeruginosa* percentage of growth reduction (%Gr) and MIC_{90} values of ZnO nanoparticles alone and doped with 2.5–10% Mn

	4.7 µg	9.4 µg	18.8 µg	37.5 µg	75 µg	150 µg	300 µg	600 µg
**$Zn_{100\%}$**	7.8%	8.4%	18.7%	45.2%	60.1%	81.9%	**100%**	100%
**$Zn_{97.5\%}Mn_{2.5\%}$**	10%	21.3%	42.2%	57.6%	75.9%	***94.6%**	100%	100%
**$Zn_{95\%}Mn_{5\%}$**	10.5%	24.6%	45.3%	59.8%	82.2%	***94.7%**	100%	100%
**$Zn_{92.5\%}Mn_{7.5\%}$**	10.8%	29.1%	47.6%	64.7%	86.8%	***97.6%**	100%	100%
**$Zn_{90\%}Mn_{10\%}$**	15.3%	35.3%	55.3%	77.7%	***92.3%**	99.2%	100%	100%

* Inhibition values considered for the determination of the Minimum Inhibitory Concentration 90% (MIC_{90})

Table S3: *S. aureus* percentage of biofilm reduction (%Br) and $MBIC_{90}$ values of ZnO nanoparticles alone and doped with 2.5–10% Mn

	37.5 µg	75 µg	150 µg	300 µg	600 µg
**$Zn_{100\%}$**	4.7%	27.6%	***100%**	100%	100%
**$Zn_{97.5\%}Mn_{2.5\%}$**	10.4%	30.1%	***100%**	100%	100%
**$Zn_{95\%}Mn_{5\%}$**	6.8%	30.5%	***100%**	100%	100%
**$Zn_{92.5\%}Mn_{7.5\%}$**	10%	34.2%	***100%**	100%	100%
**$Zn_{90\%}Mn_{10\%}$**	10.4%	35.8%	***100%**	100%	100%

* Inhibition values considered for the determination of the Minimum Biofilm Inhibitory Concentration 90% (MIC_{90})

Table S4: *P. aeruginosa* percentage of biofilm reduction (%Br) and $MBIC_{90}$ values of ZnO nanoparticles alone and doped with 2.5–10% Mn

	37.5 µg	75 µg	150 µg	300 µg	600 µg
**$Zn_{100\%}$**	3.4%	3.2%	69.3%	***100%**	100%
**$Zn_{97.5\%}Mn_{2.5\%}$**	4.4%	4.4%	70.3%	***100%**	100%
**$Zn_{95\%}Mn_{5\%}$**	2%	4.6%	71.8%	***100%**	100%
**$Zn_{92.5\%}Mn_{7.5\%}$**	8.7%	10.5%	79.6%	***100%**	100%
**$Zn_{90\%}Mn_{10\%}$**	15.3%	22.1%	***91.7%**	100%	100%

* Inhibition values considered for the determination of the Minimum Biofilm Inhibitory Concentration 90% (MIC_{90})

Table S5. Tukey's multiple-comparison analysis of *S. aureus* biofilm metabolic activity (XTT assay, OD_{490}) following treatment with 75 µg/mL of pure ZnO nanoparticles or ZnO nanoparticles doped with 2.5–10% Mn.

Condition	Mean Diff,	95,00% CI of diff,	Significant?	Summary	Adjusted P Value
CTR vs. Zn100%	0,2250	0,1381 to 0,3119	Yes	***	<0,0001
CTR vs. Zn97.5%Mn2.5%	0,2450	0,1581 to 0,3319	Yes	***	<0,0001
CTR vs. Zn95%Mn5%	0,2483	0,1614 to 0,3352	Yes	***	<0,0001
CTR vs. Zn92.5%Mn7.5%	0,2783	0,1914 to 0,3652	Yes	***	<0,0001
CTR vs. Zn90%Mn10%	0,2917	0,2048 to 0,3786	Yes	***	<0,0001
Zn100% vs. Zn97.5%Mn2.5%	0,02000	-0,07289 to 0,1129	No	ns	0,9768

Zn100% vs. Zn95%Mn5%	0,02333	-0,06955 to 0,1162	No	ns	0,9557
Zn100% vs. Zn92.5%Mn7.5%	0,05333	-0,03955 to 0,1462	No	ns	0,4413
Zn100% vs. Zn90%Mn10%	0,06667	-0,02622 to 0,1596	No	ns	0,2319
Zn97.5%Mn2.5% vs. Zn95%Mn5%	0,003333	-0,08955 to 0,09622	No	ns	>0,9999
Zn97.5%Mn2.5% vs. Zn92.5%Mn7.5%	0,03333	-0,05955 to 0,1262	No	ns	0,8340
Zn97.5%Mn2.5% vs. Zn90%Mn10%	0,04667	-0,04622 to 0,1396	No	ns	0,5741
Zn95%Mn5% vs. Zn92.5%Mn7.5%	0,03000	-0,06289 to 0,1229	No	ns	0,8843
Zn95%Mn5% vs. Zn90%Mn10%	0,04333	-0,04955 to 0,1362	No	ns	0,6431
Zn92.5%Mn7.5% vs. Zn90%Mn10%	0,01333	-0,07955 to 0,1062	No	ns	0,9962

Table S6. Tukey's multiple-comparison analysis of *S. aureus* biofilm metabolic activity (XTT assay, OD_{490}) following treatment with 37.5 µg/mL of pure ZnO nanoparticles or ZnO nanoparticles doped with 2.5–10% Mn.

**Condition**	**Mean Diff,**	**95,00% CI of diff,**	**Significant?**	**Summary**	**Adjusted P Value**
CTR vs. Zn100%	0,03833	-0,05334 to 0,1300	No	ns	0,7341
CTR vs. Zn97.5%Mn2.5%	0,08500	-0,006678 to 0,1767	No	ns	0,0758
CTR vs. Zn95%Mn5%	0,05500	-0,03668 to 0,1467	No	ns	0,3973
CTR vs. Zn92.5%Mn7.5%	0,08167	-0,01001 to 0,1733	No	ns	0,0929
CTR vs. Zn90%Mn10%	0,08500	-0,006678 to 0,1767	No	ns	0,0758
Zn100% vs. Zn97.5%Mn2.5%	0,04667	-0,05134 to 0,1447	No	ns	0,6246
Zn100% vs. Zn95%Mn5%	0,01667	-0,08134 to 0,1147	No	ns	0,9918
Zn100% vs. Zn92.5%Mn7.5%	0,04333	-0,05467 to 0,1413	No	ns	0,6896
Zn100% vs. Zn90%Mn10%	0,04667	-0,05134 to 0,1447	No	ns	0,6246
Zn97.5%Mn2.5% vs. Zn95%Mn5%	-0,03000	-0,1280 to 0,06801	No	ns	0,9048
Zn97.5%Mn2.5% vs. Zn92.5%Mn7.5%	-0,003333	-0,1013 to 0,09467	No	ns	>0,9999
Zn97.5%Mn2.5% vs. Zn90%Mn10%	0,000	-0,09801 to 0,09801	No	ns	>0,9999
Zn95%Mn5% vs. Zn92.5%Mn7.5%	0,02667	-0,07134 to 0,1247	No	ns	0,9391
Zn95%Mn5% vs. Zn90%Mn10%	0,03000	-0,06801 to 0,1280	No	ns	0,9048
Zn92.5%Mn7.5% vs. Zn90%Mn10%	0,003333	-0,09467 to 0,1013	No	ns	>0,9999

Table S7. Tukey's multiple-comparison analysis of *Pseudomonas aeruginosa* biofilm metabolic activity (XTT assay, OD_{490}) following treatment with 150 µg/mL of pure ZnO nanoparticles or ZnO nanoparticles doped with 2.5–10% Mn.

**Condition**	**Mean Diff,**	**95,00% CI of diff,**	**Significant?**	**Summary**	**Adjusted P Value**
CTR vs. Zn100%	0,9500	0,8699 to 1,030	Yes	***	<0,0001
CTR vs. Zn97.5%Mn2.5%	0,9633	0,8832 to 1,043	Yes	***	<0,0001
CTR vs. Zn95%Mn5%	0,9833	0,9032 to 1,063	Yes	***	<0,0001
CTR vs. Zn92.5%Mn7.5%	1,090	1,010 to 1,170	Yes	***	<0,0001
CTR vs. Zn90%Mn10%	1,257	1,177 to 1,337	Yes	***	<0,0001
Zn100% vs. Zn97.5%Mn2.5%	0,01333	-0,07229 to 0,09896	No	ns	0,9945
Zn100% vs. Zn95%Mn5%	0,03333	-0,05229 to 0,1190	No	ns	0,7848
Zn100% vs. Zn92.5%Mn7.5%	0,1400	0,05438 to 0,2256	Yes	**	0,0013

Zn100% vs. Zn90%Mn10%	0,3067	0,2210 to 0,3923	Yes	***	<0,0001
Zn97.5%Mn2.5% vs. Zn95%Mn5%	0,02000	-0,06562 to 0,1056	No	ns	0,9672
Zn97.5%Mn2.5% vs. Zn92.5%Mn7.5%	0,1267	0,04104 to 0,2123	Yes	**	0,0031
Zn97.5%Mn2.5% vs. Zn90%Mn10%	0,2933	0,2077 to 0,3790	Yes	***	<0,0001
Zn95%Mn5% vs. Zn92.5%Mn7.5%	0,1067	0,02104 to 0,1923	Yes	*	0,0119
Zn95%Mn5% vs. Zn90%Mn10%	0,2733	0,1877 to 0,3590	Yes	***	<0,0001
Zn92.5%Mn7.5% vs. Zn90%Mn10%	0,1667	0,08104 to 0,2523	Yes	***	0,0002

Table S8. Tukey's multiple-comparison analysis of *Pseudomonas aeruginosa* biofilm metabolic activity (XTT assay, OD_{490}) following treatment with 75 µg/mL of pure ZnO nanoparticles or ZnO nanoparticles doped with 2.5–10% Mn.

Condition	Mean Diff,	95,00% CI of diff,	Significant?	Summary	Adjusted P Value
CTR vs. Zn100%	0,04333	-0,03123 to 0,1179	No	ns	0,4291
CTR vs. Zn97.5%Mn2.5%	0,06000	-0,01456 to 0,1346	No	ns	0,1485
CTR vs. Zn95%Mn5%	0,06333	-0,01123 to 0,1379	No	ns	0,1167
CTR vs. Zn92.5%Mn7.5%	0,1433	0,06877 to 0,2179	Yes	***	0,0003
CTR vs. Zn90%Mn10%	0,3033	0,2288 to 0,3779	Yes	***	<0,0001
Zn100% vs. Zn97.5%Mn2.5%	0,01667	-0,06304 to 0,09638	No	ns	0,9795
Zn100% vs. Zn95%Mn5%	0,02000	-0,05971 to 0,09971	No	ns	0,9560
Zn100% vs. Zn92.5%Mn7.5%	0,1000	0,02029 to 0,1797	Yes	*	0,0113
Zn100% vs. Zn90%Mn10%	0,2600	0,1803 to 0,3397	Yes	***	<0,0001
Zn97.5%Mn2.5% vs. Zn95%Mn5%	0,003333	-0,07638 to 0,08304	No	ns	>0,9999
Zn97.5%Mn2.5% vs. Zn92.5%Mn7.5%	0,08333	0,003624 to 0,1630	Yes	*	0,0384
Zn97.5%Mn2.5% vs. Zn90%Mn10%	0,2433	0,1636 to 0,3230	Yes	***	<0,0001
Zn95%Mn5% vs. Zn92.5%Mn7.5%	0,08000	0,0002905 to 0,1597	Yes	*	0,0490
Zn95%Mn5% vs. Zn90%Mn10%	0,2400	0,1603 to 0,3197	Yes	***	<0,0001
Zn92.5%Mn7.5% vs. Zn90%Mn10%	0,1600	0,08029 to 0,2397	Yes	***	0,0002

Table S9. Tukey's multiple-comparison analysis of *Pseudomonas aeruginosa* biofilm metabolic activity (XTT assay, OD_{490}) following treatment with 37.5 µg/mL of pure ZnO nanoparticles or ZnO nanoparticles doped with 2.5–10% Mn.

Condition	Mean Diff,	95,00% CI of diff,	Significant?	Summary	Adjusted P Value
CTR vs. Zn100%	0,04667	-0,02543 to 0,1188	No	ns	0,3233
CTR vs. Zn97.5%Mn2.5%	0,06000	-0,01209 to 0,1321	No	ns	0,1281
CTR vs. Zn95%Mn5%	0,02667	-0,04543 to 0,09876	No	ns	0,8168
CTR vs. Zn92.5%Mn7.5%	0,1200	0,04791 to 0,1921	Yes	**	0,0011
CTR vs. Zn90%Mn10%	0,2100	0,1379 to 0,2821	Yes	***	<0,0001
Zn100% vs. Zn97.5%Mn2.5%	0,01333	-0,06374 to 0,09041	No	ns	0,9911
Zn100% vs. Zn95%Mn5%	-0,02000	-0,09707 to 0,05707	No	ns	0,9496
Zn100% vs. Zn92.5%Mn7.5%	0,07333	-0,003738 to 0,1504	No	ns	0,0660
Zn100% vs. Zn90%Mn10%	0,1633	0,08626 to 0,2404	Yes	***	0,0001
Zn97.5%Mn2.5% vs. Zn95%Mn5%	-0,03333	-0,1104 to 0,04374	No	ns	0,7076

Zn97.5%Mn2.5% vs. Zn92.5%Mn7.5%	0,06000	-0,01707 to 0,1371	No	ns	0,1704
Zn97.5%Mn2.5% vs. Zn90%Mn10%	0,1500	0,07293 to 0,2271	Yes	***	0,0002
Zn95%Mn5% vs. Zn92.5%Mn7.5%	0,09333	0,01626 to 0,1704	Yes	*	0,0146
Zn95%Mn5% vs. Zn90%Mn10%	0,1833	0,1063 to 0,2604	Yes	***	<0,0001
Zn92.5%Mn7.5% vs. Zn90%Mn10%	0,09000	0,01293 to 0,1671	Yes	*	0,0188

Table S10. Tukey's multiple-comparison analysis of *Pseudomonas aeruginosa* pyocyanin production (OD_{405}) following treatment with 150 µg/mL of pure ZnO nanoparticles or ZnO nanoparticles doped with 2.5–10% Mn.

Condition	Mean Diff,	95,00% CI of diff,	Significant?	Summary	Adjusted P Value
CTR vs. Zn100%	0,8367	0,7664 to 0,9069	Yes	***	<0,0001
CTR vs. Zn97.5%Mn2.5%	0,8667	0,7964 to 0,9369	Yes	***	<0,0001
CTR vs. Zn95%Mn5%	0,9700	0,8998 to 1,040	Yes	***	<0,0001
CTR vs. Zn92.5%Mn7.5%	1,043	0,9731 to 1,114	Yes	***	<0,0001
CTR vs. Zn90%Mn10%	1,287	1,216 to 1,357	Yes	***	<0,0001
Zn100% vs. Zn97.5%Mn2.5%	0,03000	-0,04022 to 0,1002	No	ns	0,7074
Zn100% vs. Zn95%Mn5%	0,1333	0,06311 to 0,2036	Yes	***	0,0004
Zn100% vs. Zn92.5%Mn7.5%	0,2067	0,1364 to 0,2769	Yes	***	<0,0001
Zn100% vs. Zn90%Mn10%	0,4500	0,3798 to 0,5202	Yes	***	<0,0001
Zn97.5%Mn2.5% vs. Zn95%Mn5%	0,1033	0,03311 to 0,1736	Yes	**	0,0035
Zn97.5%Mn2.5% vs. Zn92.5%Mn7.5%	0,1767	0,1064 to 0,2469	Yes	***	<0,0001
Zn97.5%Mn2.5% vs. Zn90%Mn10%	0,4200	0,3498 to 0,4902	Yes	***	<0,0001
Zn95%Mn5% vs. Zn92.5%Mn7.5%	0,07333	0,003114 to 0,1436	Yes	*	0,0389
Zn95%Mn5% vs. Zn90%Mn10%	0,3167	0,2464 to 0,3869	Yes	***	<0,0001
Zn92.5%Mn7.5% vs. Zn90%Mn10%	0,2433	0,1731 to 0,3136	Yes	***	<0,0001

Table S11. Tukey's multiple-comparison analysis of *Pseudomonas aeruginosa* pyocyanin production (OD_{405}) following treatment with 75 µg/mL of pure ZnO nanoparticles or ZnO nanoparticles doped with 2.5–10% Mn.

Condition	Mean Diff,	95,00% CI of diff,	Significant?	Summary	Adjusted P Value
CTR vs. Zn100%	0,5200	0,4060 to 0,6340	Yes	***	<0,0001
CTR vs. Zn97.5%Mn2.5%	0,6300	0,5160 to 0,7440	Yes	***	<0,0001
CTR vs. Zn95%Mn5%	0,6200	0,5060 to 0,7340	Yes	***	<0,0001
CTR vs. Zn92.5%Mn7.5%	0,7333	0,6193 to 0,8473	Yes	***	<0,0001
CTR vs. Zn90%Mn10%	0,8633	0,7493 to 0,9773	Yes	***	<0,0001
Zn100% vs. Zn97.5%Mn2.5%	0,1100	-0,003998 to 0,2240	No	ns	0,0609
Zn100% vs. Zn95%Mn5%	0,1000	-0,01400 to 0,2140	No	ns	0,0988
Zn100% vs. Zn92.5%Mn7.5%	0,2133	0,09934 to 0,3273	Yes	***	0,0004
Zn100% vs. Zn90%Mn10%	0,3433	0,2293 to 0,4573	Yes	***	<0,0001
Zn97.5%Mn2.5% vs. Zn95%Mn5%	-0,01000	-0,1240 to 0,1040	No	ns	0,9996
Zn97.5%Mn2.5% vs. Zn92.5%Mn7.5%	0,1033	-0,01066 to 0,2173	No	ns	0,0842

Zn97.5%Mn2.5% vs. Zn90%Mn10%	0,2333	0,1193 to 0,3473	Yes	***	0,0002
Zn95%Mn5% vs. Zn92.5%Mn7.5%	0,1133	-0,0006649 to 0,2273	No	ns	0,0517
Zn95%Mn5% vs. Zn90%Mn10%	0,2433	0,1293 to 0,3573	Yes	***	0,0001
Zn92.5%Mn7.5% vs. Zn90%Mn10%	0,1300	0,01600 to 0,2440	Yes	*	0,0226

Table S12. Tukey's multiple-comparison analysis of *Pseudomonas aeruginosa* pyocyanin production (OD_{405}) following treatment with 37.5 μg/mL of pure ZnO nanoparticles or ZnO nanoparticles doped with 2.5–10% Mn.

**Condition**	**Mean Diff,**	**95,00% CI of diff,**	**Significant?**	**Summary**	**Adjusted P Value**
CTR vs. Zn100%	0,1833	0,09055 to 0,2761	Yes	***	0,0003
CTR vs. Zn97.5%Mn2.5%	0,2233	0,1306 to 0,3161	Yes	***	<0,0001
CTR vs. Zn95%Mn5%	0,2700	0,1772 to 0,3628	Yes	***	<0,0001
CTR vs. Zn92.5%Mn7.5%	0,2400	0,1472 to 0,3328	Yes	***	<0,0001
CTR vs. Zn90%Mn10%	0,3500	0,2572 to 0,4428	Yes	***	<0,0001
Zn100% vs. Zn97.5%Mn2.5%	0,04000	-0,05278 to 0,1328	No	ns	0,7000
Zn100% vs. Zn95%Mn5%	0,08667	-0,006113 to 0,1794	No	ns	0,0723
Zn100% vs. Zn92.5%Mn7.5%	0,05667	-0,03611 to 0,1494	No	ns	0,3707
Zn100% vs. Zn90%Mn10%	0,1667	0,07389 to 0,2594	Yes	***	0,0006
Zn97.5%Mn2.5% vs. Zn95%Mn5%	0,04667	-0,04611 to 0,1394	No	ns	0,5621
Zn97.5%Mn2.5% vs. Zn92.5%Mn7.5%	0,01667	-0,07611 to 0,1094	No	ns	0,9887
Zn97.5%Mn2.5% vs. Zn90%Mn10%	0,1267	0,03389 to 0,2194	Yes	**	0,0064
Zn95%Mn5% vs. Zn92.5%Mn7.5%	-0,03000	-0,1228 to 0,06278	No	ns	0,8778
Zn95%Mn5% vs. Zn90%Mn10%	0,08000	-0,01278 to 0,1728	No	ns	0,1071
Zn92.5%Mn7.5% vs. Zn90%Mn10%	0,1100	0,01722 to 0,2028	Yes	*	0,0175
